# A Cradle‐to‐Gate Life Cycle Assessment of Sustainable All‐Cellulose Composites Using Imidazolium‐Based Ionic Liquid

**DOI:** 10.1002/gch2.70167

**Published:** 2026-09-30

**Authors:** Guanyi Chen, Keeran Ward, Daniel L. Baker, Diego Freire Ordóñez, Michael E. Ries

**Affiliations:** ^1^ School of Physics and Astronomy University of Leeds Leeds UK; ^2^ School of Chemical and Process Engineering (SCAPE) University of Leeds Leeds UK; ^3^ School of Mathematics University of Leeds Leeds UK; ^4^ Department of Chemical Engineering Imperial College London London UK

**Keywords:** 1‐ethylimidazolium acetate, all‐cellulose composite, ionic liquid, life cycle assessment, sustainability, textile

## Abstract

All‐cellulose composites (ACCs) are single‐component bio‐based green composites made from cellulose, enabling simplified recycling because ACCs have no chemical incompatibility between their matrix and reinforcement fibers. Yet, few life cycle assessment (LCA) studies have examined environmental performance of ACCs. This study assessed the ACC sustainability through a cradle‐to‐gate LCA following the ISO 14040: 2006 framework. ACCs were produced via cellulose dissolution in either stand‐alone ionic liquid 1‐ethyl‐3‐methylimidazolium acetate (EMIM OAc) or with its co‐solvent dimethyl sulfoxide (DMSO). DMSO served as a co‐solvent to reduce the EMIM OAc viscosity and improve cellulose dissolution. The E60D40 solvent (60 wt.% EMIM OAc, 40 wt.% DMSO) exhibited the lowest global warming potential (GWP) at 0.25 kg CO_2_ eq/FU, whereas the E20D80 had the highest at 0.75 kg CO_2_ eq/FU, exceeding stand‐alone EMIM OAc (0.33 kg CO_2_ eq/FU), where FU is the equivalent mass of ACCs. Human health, ecosystem quality, and resource scarcity impacts ranged from 5.06 × 10^−7^–1.56 × 10^−6^ DALY/FU, 1.15 × 10^−9^–3.94 × 10^−9^ species.yr/FU, and 2.70 × 10^−2^–8.20 × 10^−2^ USD_2013_/FU, respectively across different solvent scenarios. These findings illustrate that ACC development can be made more sustainable with DMSO's influence over pure EMIM OAc type solvent formulations.

## Graphical abstract

All‐cellulose composites produced from cotton textiles were evaluated for sustainability and mechanical performance. Adding dimethyl sulfoxide (DMSO) to the ionic liquid 1‐ethyl‐3‐methylimidazolium acetate (EMIM OAc) reduced environmental burdens while preserving high stiffness. The solvent formulation containing 60 wt.% EMIM OAc and 40 wt.% DMSO delivered the balance of sustainability and performance, reducing CO_2_ emissions by 24% versus pure EMIM OAc.

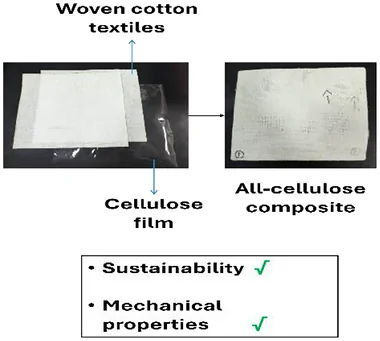

## 1 Introduction

There is an increased demand for developing composite materials from renewable biobased materials to meet the need for sustainable growth, energy security, and a lower carbon footprint [1]. Challenges faced by traditional composite materials, e.g., carbon fiber‐epoxy resin composites, include end‐of‐life recycling, the nature of predominantly being fossil fuel‐ based, and the high global warming potential (GWP) of the composite manufacture [1, 2, 3]. For example, the GWP of producing 1 kg carbon fiber‐based composites (CFC) is 54 kg CO_2_ [3]. Most of the composite materials have glass, aramid, or carbon fibers as their reinforcement fibers and thermoplastics as the matrix [1]. These composites tend to end up in landfill or incineration because of the difficulty in separating the matrix and reinforcement fiber for recycling [1].

Cellulose is a renewable and biodegradable biopolymer that can be found in species of plants, algae, fungi, and bacteria [4]. It is estimated that global cellulose production is about 1.5 × 10^12^ tons per year [4]. There are four cellulose allomorphs (I‐IV). Cellulose I is the native cellulose that naturally exists, while the rest of the allomorphs are based on modifications of cellulose I. Cellulose I is found to have the strongest mechanical properties among all cellulose allomorphs. Its theoretical ultimate tensile strength is 13–17 GPa [4]. In comparison, cellulose II has a tensile strength of about 9 GPa [4]. The Young's modulus (YM) of cellulose I can reach 138 GPa, which is higher than the YM of aluminum (70 GPa) or glass fibers (76 GPa) [4]. The competitive mechanical properties of cellulose I make it attractive for composite applications.

An ACC is a composite where its matrix and reinforcement fibers are both made of cellulose, meaning there is no chemical incompatibility between its matrix and reinforcement fibers [4]. The matrix and the reinforcement fibers may contain chemically similar cellulosic materials [4]. For example, the matrix of ACC can be cellulose I, while the reinforcement fibers are cellulose II. Since cellulose does not melt, the matrix of an ACC is formed through cellulose dissolution in solvents of ionic liquids (ILs) like EMIM OAc, optionally with co‐solvents, i.e., DMSO [5]. DMSO is a colorless liquid formed as a by‐product in the production of paper [6]. DMSO is found to improve the solvation ability of EMIM OAc through mass transport, decreasing the viscosity of EMIM OAc significantly and hence increases the efficiency of cellulose dissolution in EMIM OAc [7].

ACCs can be produced through either a two‐step method, or a one‐step method [4]. In the two‐step method, a solution of dissolved cellulose acts as the matrix, which is added to the chosen cellulosic reinforcement fibers to form an ACC [8]. In the one‐step method, cellulosic materials are partially dissolved by solvents [9]. The partially dissolved cellulosic surface becomes the matrix of the ACC and forms around the undissolved cellulose that works as the reinforcement fibers [4, 9]. Because both the matrix and the reinforcement fibers of an ACC are made of renewable cellulosic materials, ACC can be recycled simply without matrix and fiber separation through cellulose dissolution [10]. This gives ACCs a high potential to replace traditional fossil fuel‐based composites and to form a circular economy in the composite industry because the dissolved cellulose can be reused as a cellulose source to produce new ACCs [4, 11, 12].

The textile and clothing industry is the second most polluting industry on Earth, taking up 8–10% of the global carbon emissions, which is higher than the carbon emissions from the aviation and shipping industries [13, 14]. The textile industry emits about 1.7 Bt CO_2_ yearly [15]. It is estimated that the industry's greenhouse gas emissions in 2030 will be about 30% higher than the level in 2022 [13, 16]. The industry is also the second largest water consumer in the world and one of the biggest pollution sources that pollute waterways by microplastics and chemicals [13]. Due to the increased demand in textiles, the amount of textile waste will also increase [17]. Brundtland [18] estimated that over 100 million new apparels were produced yearly, but 92 million of them would end up in landfill. To make the textile industry more sustainable, the industry requires better waste management and production methods [17]. Cotton textiles can be used as a cellulose source to produce ACCs since native cotton consists of 88–96.5% cellulose I, with the rest of the components being mainly proteins, pectins, and waxes [19]. Both new and post‐consumer cotton textiles can be used to make ACCs, and the resulting YM was shown to be above 3 GPa [5, 19, 20]. Therefore, ACCs have the potential to be a solution to textile waste management, possibly contributing to the formation of a circular economy in the textile industry.

Despite some literature [4, 5, 19, 21, 22] suggesting that ACCs are sustainable alternatives to traditional fossil fuel‐based composites, having the potential to reduce the environmental impacts in the composite and textile industries, there is limited quantitative research about the sustainability of ACC and its environmental impact [23]. Therefore, a cradle‐to‐gate life cycle assessment (LCA) was constructed in this study to evaluate the environmental impacts associated with a specific ACC production process in a laboratory scale, following the ISO 14040: 2006 LCA framework [24]. The mechanical properties and environmental impacts of ACCs made of woven cotton textiles from various solvent mixtures were studied to understand the sustainability of ACCs and the trade‐off between their sustainability and mechanical performance under the investigated cradle‐to‐gate ACC production system.

## 2 Methods

### 2.1 Production of All‐Cellulose Composites

#### 2.1.1 Materials

100% bleached cotton woven fabrics (Oh Sew Crafty, Kidderminster, UK), and Natureflex 23NP cellulose films (Futamura Chemical UK, Ltd., Cumbria, UK) were the cellulose sources. EMIM OAc (proionic GmbH) with a purity of ≥98%, and its co‐solvent DMSO (fluorochem) with a purity of 99% were used as solvents in this study.

#### 2.1.2 ACC Preparation

ACC samples were made based on the method published in patent EP3823823B1 [25] and Victoria et al. [5], where an interleaf cellulose film was used (Figure 1). No conditioning or pre‐treatment was done to either the cotton textiles or the cellulose films prior to the ACC production. Each ACC sample consisted of two cotton textiles and one cellulose film. Bleached cotton textiles and cellulose films were cut into a size of 10 cm × 10 cm. Cotton textiles were aligned in their warp direction (0°, 0°) orientation, with the cellulose film sandwiched in between. Solvents consisted of various EMIM OAc wt.% and DMSO wt.% were made (Table 1). The mass ratio between the solvent and the cotton‐cellulose film stack was 3:1 to compensate for the flash during the dissolution process under the hot press to provide sufficient cellulose dissolution [5]. The solvent mixture of EMIM OAc and DMSO was applied to each layer of cotton fabric and cellulose film by hand. According to Victoria et al. [20], cellulose dissolution at 100°C, 2 MPa for 100 min in an 80 wt.% EMIM OAc/20 wt.% DMSO solvent can maximize the YM of ACCs to 3.2 GPa. Therefore, these processing conditions were used. The sample was pressed in a mould at 100°C, 2 MPa for 100 min for cellulose dissolution and heat compaction to maximize the YM. After hot pressing, the sample was moved to a bath of tap water (coagulant), immersed in water for 24 ± 1 h to wash out the remaining solvent. For every 10 g of solvent used, at least 2.5 L of tap water was added to the water bath. After coagulation in the water bath, the ACC sample was rinsed under running tap water and then dried at 110°C, 2 MPa for 60 min.

**FIGURE 1:**
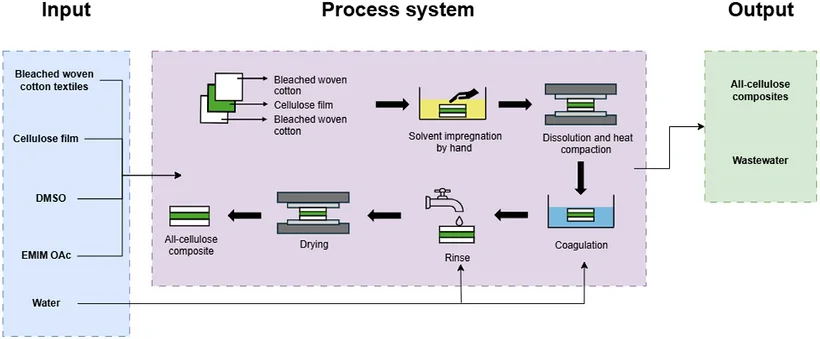
The flow chart of ACC preparation with system boundaries.

**TABLE 1:** Composition of examined solvents.

Solvent	EMIM OAc wt.%	DMSO wt.%
**E100**	100	0
**E80D20**	80	20
**E60D40**	60	40
**E40D60**	40	60
**E20D80**	20	80

### 2.2 Mechanical Testing

Based on the ASTM D638 standard, the resulting ACC samples were laser‐cut into type V dumbbell specimens to obtain YM, using an Instron 5584 universal tensile tester. The crosshead speed was set as 1 mm/min. Five dumbbell specimens were cut and used for tensile testing on the ACC sample obtained from each solvent system. The gradient at 0.5% strain on the stress‐strain curve for each specimen was used to obtain its corresponding YM.

#### 2.2.1 Statistical Analysis

To evaluate whether the YMs of ACCs made in the five solvent scenarios can be distinguished, a one‐way ANOVA was performed using the raw values of YM from the five dumbbell specimens for each solvent formulation in Excel. The raw Excel data is provided in the ESI.

### 2.3 ACC Density Calculations

The resulting ACC sample was laser cut in a 7.00 cm × 8.60 cm rectangle. The mass of the rectangle was measured by a balance, and the thickness of the rectangle at 24 points was measured by a digital caliper (Onecall, Farnell) to obtain the average thickness of the sample.

### 2.4 Synthesis of Ionic Liquid

EMIM OAc purchased from proionic GmbH is produced by carbonate based ionic liquid synthesis (CBILS), which is a process free from halogen based and carcinogenic chemicals [26]. The full CBILS process includes two alkylation reactions and cation exchange [26]. The first step of alkylation produces 1‐alkylimidazole as a precursor, and by‐products of CO_2_ and alcohol (Equation 1). The second step of alkylation produces the intermediate of the aimed ionic liquid (Equation 2). An alcohol, e.g., methanol, can be used as a catalyst in this step. The final step of cation exchange converts the intermediate to the aimed ionic liquid by the addition of Brønsted acid, producing CO_2_ and alcohol as by‐products (Equation 3).

(1) $$C_{3}H_{4}N_{2}+R_{2}{\mathrm{CO}}_{3}\to {\mathrm{RC}}_{3}H_{3}N_{2}+{\mathrm{CO}}_{2}+\mathrm{ROH}$$

(2) $$RC_{3}H_{3}N_{2}+{R^{\prime }}_{2}CO_{3}\to {\left[C_{n}C_{m}\mathrm{im}\right]}^{+}{\left[R^{\prime }CO_{3}\right]}^{-}+CO_{2}+R^{\prime }\mathrm{OH}\;$$

(3) $${\left[C_{n}C_{m}\mathrm{im}\right]}^{+}{\left[R^{\prime }CO_{3}\right]}^{-}+\mathrm{HA}\to {\left[C_{n}C_{m}\mathrm{im}\right]}^{+}{\left[A\right]}^{-}+CO_{2}+R^{\prime }\mathrm{OH}\;$$

The process flowsheet of EMIM OAc production with system boundaries is shown in Figure 2. The process was derived by mass and energy balances utilizing specific heat capacity data on EMIM OAc obtained from differential scanning calorimetry (DSC) on EMIM OAc. The specific heat capacity equation of EMIM OAc was estimated from the DSC experiments, and the corresponding details on the DSC method are in Section S1.

**FIGURE 2:**
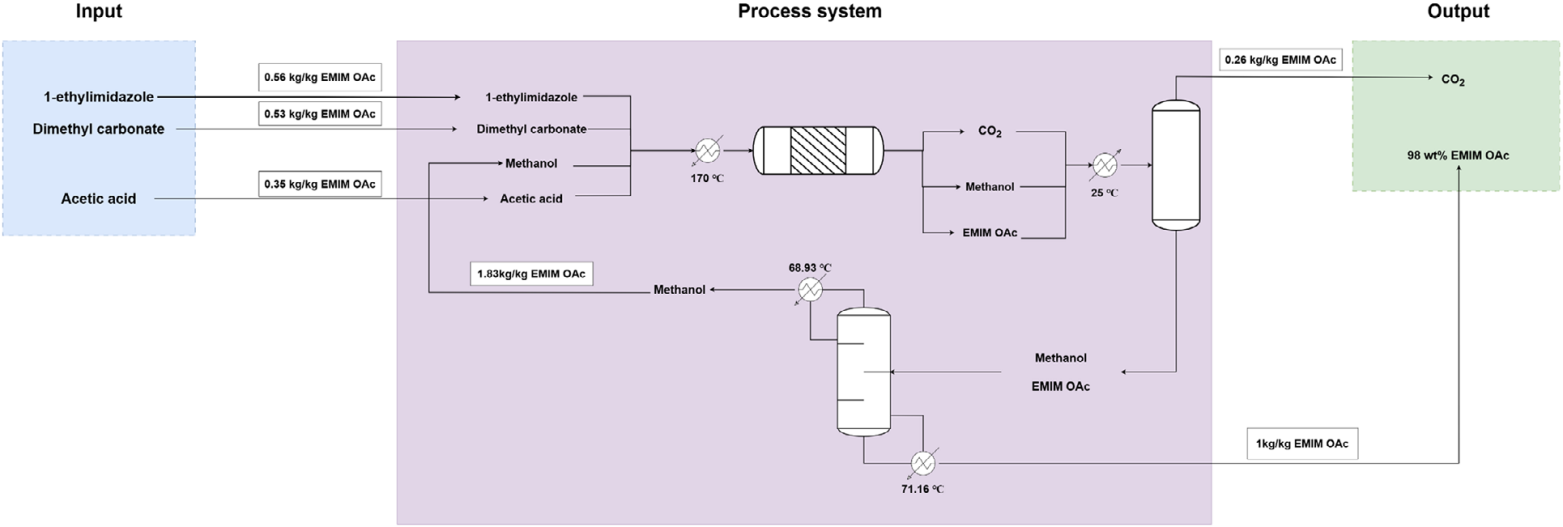
The process flow chart of EMIM OAc with system boundaries.

According to the manufacturer, the EMIM OAc (proionic GmbH) production started with 1‐ethylimidazole imported from Mainland China with the CBILS process carried out in Austria (Figure 2). The inventories and production system boundaries for EMIM OAc were developed to represent the specific supply chain and CBILS production used by proionic GmbH. Mass and energy balances were performed over the full process flowsheet, with EMIM OAc inventories given in Sections S1 and S2.

Methanol was used as a catalyst, with an assumption of full methanol recovery and recycling within the process. 1‐ethylimidazole (C_5_H_8_N_2_), dimethyl carbonate (C_3_H_6_O_3_), acetic acid (C_2_H_4_O_2_), and methanol (CH_3_OH) were heated to 170°C from 25°C at 40 bar, forming EMIM OAc ([C_6_H_11_N_2_]^+^[C_2_H_3_O_2_]^−^) with a 100% conversion on the intermediate during reaction. After this reaction (Equation 4), the mixture of methanol, EMIM OAc, and CO_2_ were cooled down to 25°C and flash‐separated, with CO_2_ being preferentially removed. The mixture of methanol and EMIM OAc was then further separated following distillation, operating at 1.2 bar with purities of 98% and 100% for EMIM OAc and methanol, respectively.

(4) $$\begin{array}{cc} & C_{5}H_{8}N_{2}+C_{3}H_{6}O_{3}+C_{2}H_{4}O_{2}+\;CH_{3}\mathrm{OH}\;\left(\mathrm{cat}.\right)\to {\left[C_{6}H_{11}N_{2}\right]}^{+}\\ & {\left[C_{2}H_{3}O_{2}\right]}^{-}+\;CO_{2}+\;CH_{3}\mathrm{OH}\;+\;CH_{3}\mathrm{OH}\;\left(\mathrm{cat}.\right) \end{array}$$

### 2.5 Life Cycle Assessment (LCA)

#### 2.5.1 Level 1: Goal and Scope Definitions

This study aims to evaluate the cradle‐to‐gate environmental impact of ACC produced in the same manufacturing process (Figure 1) but with various solvent mixtures, consisting of different wt.% of EMIM OAc and DMSO. The recycling of EMIM OAc and DMSO was not considered in this study. ACC produced in 100 wt.% EMIM OAc with no DMSO addition was set to be the benchmark material (business‐as‐usual, BAU) for comparison.

#### 2.5.2 Level 2: Life Cycle Inventory (LCI)

The foreground system inventories were gathered from literature data, and mass and energy balances, i.e., production of EMIM OAc. The inventories for the ACC production were based on wet lab experiments of making the ACCs. The electricity consumption for the laboratory process was estimated from power measurements using an oscilloscope. Background inventory data were obtained from Ecoinvent v3 databases. The input and output of the production of ACCs in a laboratory scale is illustrated in Figure 1. The inputs consisted of woven cotton textiles, cellulose films, tap water, EMIM OAc and DMSO. The outputs consisted of the produced ACCs and wastewater. Inventories for the precursor, 1‐ethylimidazole were obtained by Zhang et al. [27]. Normalized inventories regarding the production of ACCs in each solvent system are given in Table 2. Details about the LCI entries for each solvent scenario inventory are given in Section S2 Table S1. The LCI of EMIM OAc is in Tables S1 and S12.

**TABLE 2:** Life cycle inventories for the lab‐scaled production of ACCs in five solvents (E100, E80D20, E60D40, E40D60, E20D80). Functional unit (FU) = equivalent mass of ACCs produced with respect to 2.8000 g of ACC produced in the E100 solvent.

Solvent system	E100	E80D20	E60D40	E40D60	E20D80
Inputs‐materials					
Woven cotton textiles [g]	2.9250	2.4041	2.4507	3.8377	8.5025
Cellulose film [×10^−2^ m^2^]	1.0000	0.8180	0.8520	1.2460	2.8950
Tap water [kg]	2.5000	2.1051	2.1687	3.4204	7.3424
EMIM OAc [g]	9.7500	6.4249	4.9187	5.1443	5.6683
DMSO [g]	0.0000	1.6083	3.2791	7.7027	22.7027
Inputs‐energy					
Electricity [kWh]	0.6189	0.5212	0.5369	0.8468	1.8177
Outputs‐materials					
ACC [g]	2.8000	2.2904	2.3856	3.4888	8.1060
Outputs‐emissions					
Wastewater [×^−3^ m^3^]	2.5000	2.1051	2.1687	3.4204	7.3424

##### 2.5.2.1 Functional Unit

A functional unit, i.e., mass, was used to compare environmental impacts generated by ACCs produced using various solvent mixtures. The ACCs produced do not have the same mechanical properties. To ensure compared materials have equivalent mechanical properties to fulfil comparable structural applications, a performance indicator (PI), like YM or tensile strength, is required [28, 29]. YM measures the stiffness of a material under uniaxial elastic loading and quantifies a material's resistance to elastic deformation [28]. YM was primarily aimed to be maximized in the present ACC fabrication process in comparison to other mechanical properties. Therefore, it was used as the PI to obtain the equivalent mass of the material, so the material will have the same stiffness level as the benchmark material in the same dimension [30, 31]. The equivalent mass can be calculated by the following equation reviewed by Hervey et al. [31]:

(5) $$m=m_{ref}\left(\frac{E_{ref}/\rho_{ref}}{E/\rho }\right)$$

with *m*, *E*, *ρ* represent mass, YM, and density of the chosen material, respectively. The mass, YM, and density of the reference material are represented by *m_ref_*, *E_ref_*, *ρ_ref_* respectively. In this study, the reference material is 2.80 g ACC produced with 100 wt.% EMIM OAc (E100). The equivalent masses of ACCs produced across each solvent mixture (E80D20, E60D40, E40D60, E20D80), and the mass of the reference material can be found in Table 3.

**TABLE 3:** The mechanical properties and functional units of ACCs produced in the five solvents.

Solvent	YM [GPa]	Density [kg/m^3^]	Equivalent Mass Ratio^a^	Functional Unit [g ACC]
E100 (benchmark)	4.3 ± 0.4	963 ± 51	1.000	2.8000
E80D20	5.3 ± 0.4	984 ± 67	0.818	2.2904
E60D40	5.3 ± 0.5	1014 ± 62	0.852	2.3856
E40D60	3.0 ± 0.2	855 ± 32	1.246	3.4888
E20D80	1.3 ± 0.1	876 ± 33	2.895	8.1060

^a^ The equivalent mass ratio was calculated based on the raw values of YM and density and kept in 3 decimal places for the functional unit calculation. The raw values of YM and density are provided in the ESI Excel file.

#### 2.5.3 Level 3: Life Cycle Impact Assessment (LCIA)

The environmental impact comparison among ACCs made with the five solvent mixtures is based on the equivalent stiffness instead of an overall structural performance. Both midpoint and endpoint characterization based on the ReCiPe 2016 Hierarchist (H) methods were used to evaluate the environmental impact and possible burden‐shifting of ACCs produced in each solvent, using the SimaPro v10.3.0.1 LCA software. The Hierarchist (H) perspective method was chosen for this study as it considers a balanced time perspective (100‐year), compromising both short‐term (20‐year) and long‐term (1000‐year) impacts with proven evidence accepted by international bodies like the World Health Organization and provides quantitative results in environmental burdens [32, 33, 34].

#### 2.5.4 Level 4: Interpretation

By analyzing the LCIA results across both midpoint and endpoint impact categories, together with ACC mechanical properties, multiple decision‐making criteria were developed to compare scenarios, deducing the preferred solvent formulation that provides relatively high YM while minimizing environmental impacts under the specified ACC production conditions in the present study.

A normalized spider plot consisting multiple decision‐making criteria was constructed as a visual representation of the decision‐making criteria across the scenarios. The spider plot was interpreted qualitatively, while the ranking of the scenarios was based on the sum of normalized scores.

Equal weighting was assigned to each criterion to avoid subjective bias on individual criterion and to give a transparent baseline comparison. Since different units and magnitudes were used by different criteria, maximum normalization procedure was applied to transform the criteria into dimensionless values on a common scale ranging between 0 and 1, following Equation (6) [35].

(6)

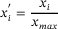

where *x_i_* ^′^ represents normalized score of criterion *i* for a given scenario, *x_i_* represents the absolute original value, and *x_max_* is the highest absolute value of the corresponding criterion among all evaluated scenarios.

Maximum normalization method was selected because this study aimed to compare the ACCs made using the five solvent formulations under the same system boundary instead of evaluating their mechanical and environmental performances against external reference systems. This approach also preserves the relative difference among scenarios with respect to a criterion. It is also important to note that the LCA‐derived formulation ranking (See Results and Discussion‐ Section 3.3) is conditional due to the absence of quantitative parameter‐sensitivity analysis. The derived formulation ranking depends on the specific inventories and boundary conditions used in the present study and is subject to change if the underlying inventory data or modeling assumptions are altered.

#### 2.5.5 Key Assumptions

Several assumptions are made in this LCA study due to the lack of accurate LCI. Here is the list of assumptions:

1. The LCI of Natureflex 23NP cellulose film is not available, so the LCIA of producing 1 m^2^ cellophane film [36, 37] was used in the analysis because Natureflex 23NP cellulose film is a cellophane film. It was assumed that 0.01 m^2^ of cellulose film was used in the reference ACC.
2. The conversion of the intermediate in the synthesis of EMIM OAc was assumed to be 100% and fast.
3. The 2% impurity in EMIM OAc was assumed to be methanol.
4. Water consumed for rinsing ACC samples was ignored in the inventory because the volume of water used for rinsing was negligible in comparison to the water used for coagulation.
5. The YM and density of the ACC samples were assumed to be the same across the sample.

## 3 Results and Discussion

The equivalent mass of ACCs produced in the five solvents, the corresponding YM, and the densities of the ACCs are shown in Table 3. The errors are standard deviations and standard errors for YM and density, respectively. Figure 5 shows the density of ACC against the wt.% of DMSO used in different solvents in this study.

### 3.1 Young's Modulus and Density

YM was used as the PI in this LCA study. Figure 3 shows the YM of ACCs made with different DMSO wt.%. The resulting YMs from all the solvents, except the E20D80 solvent, was above 3 GPa. A one‐way ANOVA test was performed across the five solvents. The results (Table 4) showed that there was a statistically significant difference among the five solvents, where F‐value = 120.77 and *p*‐value = 1.05 × 10^−13^. The F‐value was significantly higher than the critical *F*‐value, and the p‐value is significantly smaller than 0.05, meaning that the variation in YMs was not due to random experimental variations. The YM peaked at 20 wt.% DMSO, reaching 5.3 ± 0.4 GPa. The YM was 5.3 ± 0.5 GPa when the sample was made with 40 wt.% DMSO, which is very similar to the one made with 20 wt.% DMSO. Therefore, a one‐way ANOVA test was performed to determine whether YMs of ACCs produced in these two formulations can be distinguished. The results (Table 5) indicated that there was no statistically significant difference between the YMs resulted from the E80D20 and E60D40 solvents, as the F‐value = 0.04 and p‐value = 0.84. This suggests that under this ACC production setup, an optimal YM may occur when 20–40 wt.% DMSO is added to EMIM OAc. As the concentration of DMSO increased, YM decreased and reached 1.3 ± 0.1 GPa when there was 80 wt.% DMSO in the solvent. This suggests that 40 wt.% DMSO may be a critical point of cellulose dissolution in the ACC preparation. As the concentration of DMSO increases, the concentration of available anions and cations from EMIM OAc is too low to perform cellulose dissolution. Hence, there is less cellulosic matrix to hold the reinforcement fibers in the ACC to dissipate the stretching force used in the tensile test [38].

**FIGURE 3:**
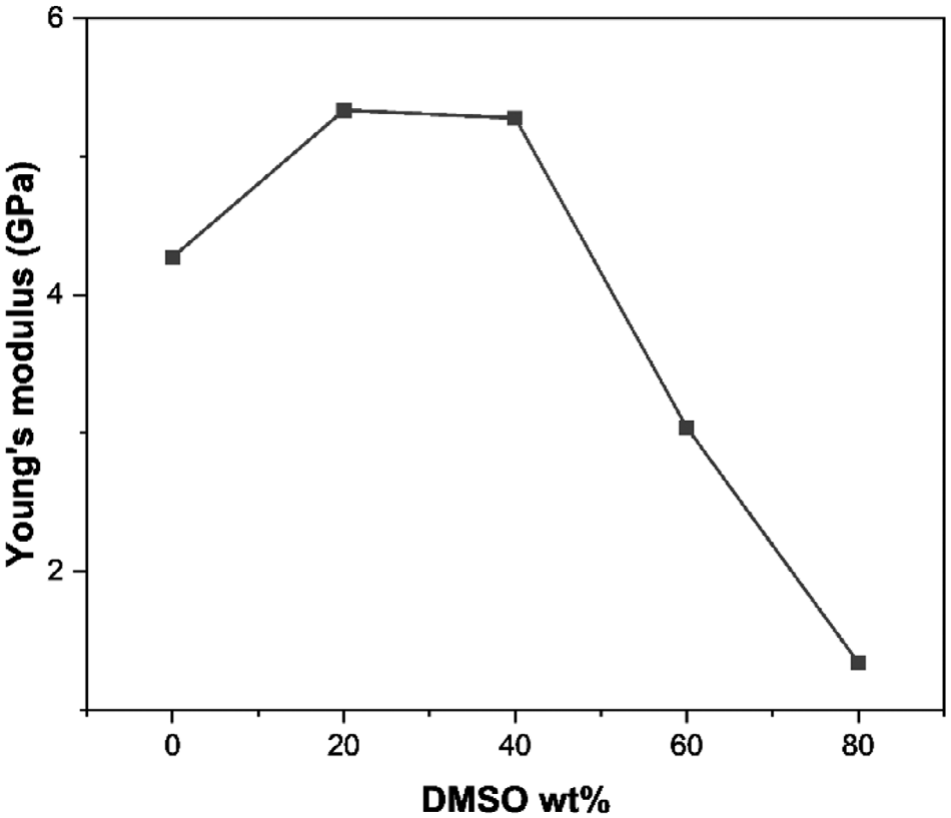
The Young's modulus of ACC produced against the wt.% of DMSO used in the solvent.

**TABLE 4:** One‐way ANOVA from the Young's modulus of ACCs made in the five solvent formulations.

Source of Variation	Sum of squares	Degrees of freedom	Mean square	F‐value	*p*‐value	Critical F value
Between Groups	56.86	4.00	14.21	120.77	1.05E‐13	2.87
Within Groups	2.35	20.00	0.12			
Total	59.21	24.00				

**TABLE 5:** One‐way ANOVA from the Young's modulus of ACCs made in the E80D20 and E60D40 solvents.

Source of Variation	Sum of squares	Degrees of freedom	Mean square	F‐value	*p*‐value	Critical F value
Between Groups	0.01	1.00	0.01	0.04	0.84	5.32
Within Groups	1.44	8.00	0.18			
Total	1.45	9.00				

Figure 4 shows the density of ACCs made with different DMSO wt.% in the solvents. The density of ACC increased from 0 wt.% to 40 wt.% DMSO. It peaked to 1014 ± 61 kg/m^3^ at 40 wt.% DMSO. When the concentration of DMSO was over 40 wt.% in the solvent, the density value dropped significantly, suggesting that 40 wt.% DMSO may be a critical point for the ACC density as well.

**FIGURE 4:**
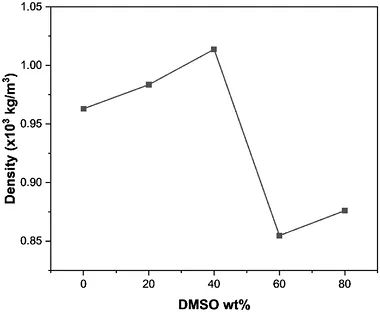
The density of ACC produced against the wt.% of DMSO used in the solvent.

### 3.2 Life Cycle Impact Assessment (LCIA)

Environmental burdens resulting from ACC production regarding each solvent were characterized using the ReCiPe 2016 midpoint Hierarchist perspective method. The details of the midpoint LCIA regarding the five solvent scenarios are shown in Section S2 Tables S2–S6. The midpoint LCIA of EMIM OAc is in Section S2.1 Table S13. Figure 5 shows the burdens of 18 impact categories, with stacked columns representing contributions across inventories. Electricity usage during the ACC lab production was the most significant hotspot across 13 out of 18 categories, namely GWP, ionizing radiation, ozone formation (HH), ozone formation (Terrestrial), PMF, terrestrial acidification, terrestrial ecotox., freshwater ecotox., marine ecotox., human carcino., human non‐carcino., mineral resources, and fossil resources. The contribution from electricity ranged from 28%–97% in the five solvent scenarios across these 13 impact categories. The lowest contribution from electricity was in the E100 scenario in human non‐carcino. The highest contribution from electricity occurred in the E20D80 scenario in ionizing radiation. These contributions are likely due to the UK grid mix, with burdens strongly linked to ionizing radiation, such as radon‐222 attributed to nuclear power. Nuclear electricity makes up about 15% of the UK's electricity in the UK energy mix [39]. The use of uranium in electricity production also contributes to mineral resources. Other significant burdens include metals such as zinc, copper, and mercury, derived from fossil fuel extraction, contribute to ecotoxicity. Furthermore, combustion and fugitive emissions derived from fossil fuel utilization [40], including CO_2_, CH_4_, N_2_O, NO_x_, particulates and SO_2,_ impact significantly across GWP, fossil resources, human health, terrestrial acidification, PMF, and ozone formation.

**FIGURE 5:**
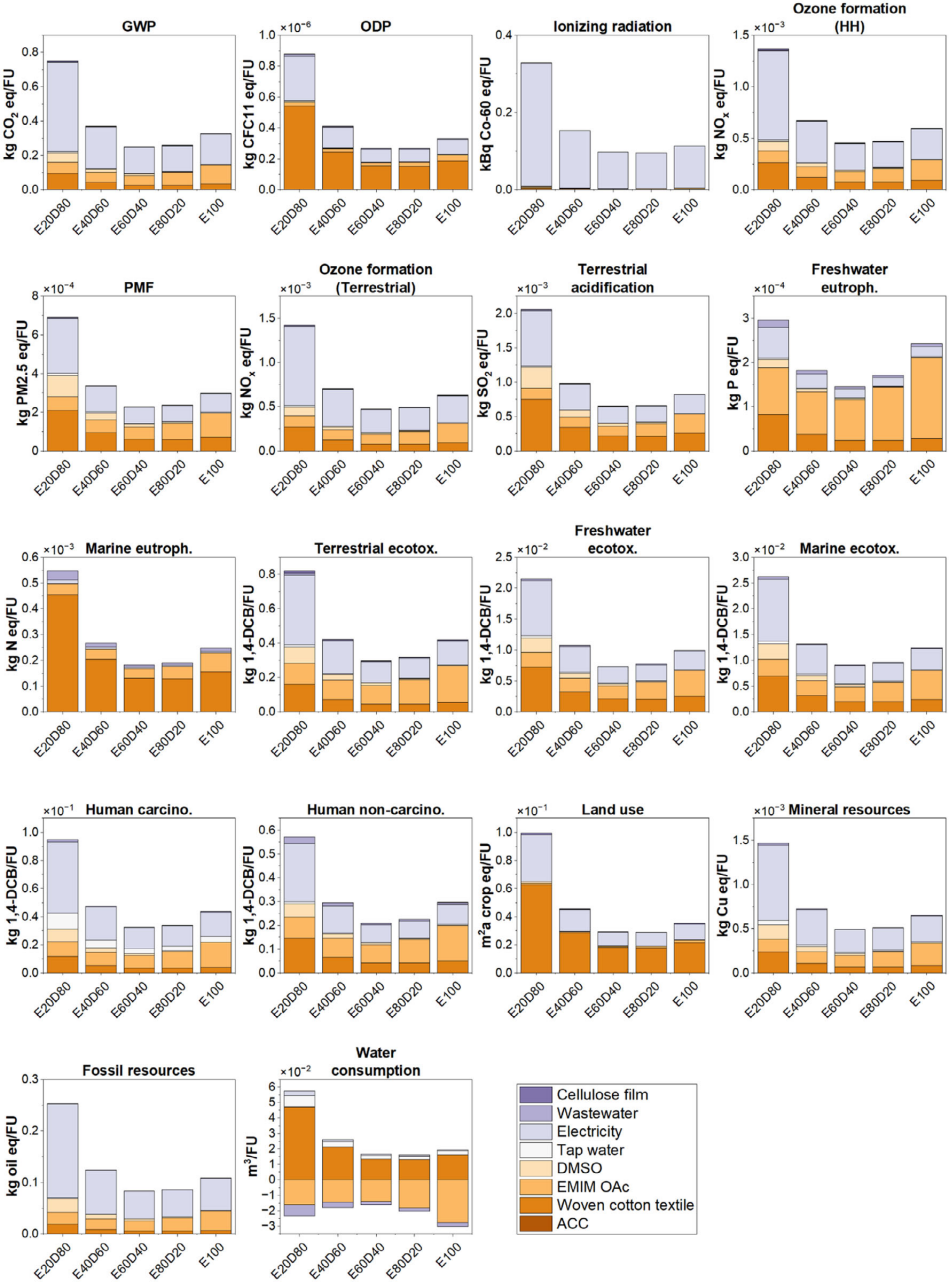
Characterized LCIA results for producing 1 functional unit of ACC in a lab scale in five different solvents across 18 midpoint impact categories assessed by the ReCiPe 2016 (H) method. GWP: global warming potential; ODP: ozone depletion; Ozone formation (HH): ozone formation (human health); PMF: fine particulate matter; Ozone formation (Terrestrial): ozone formation (terrestrial ecosystems); Terrestrial acidification; Freshwater eutroph.: freshwater eutrophication; Marine eutroph.: marine eutrophication; Terrestrial ecotox.: terrestrial ecotoxicity; Freshwater ecotox.: freshwater ecotoxicity; Marine ecotox.: marine ecotoxicity; Human carcino.: human carcinogenic toxicity; Human non‐carcino.: human non‐carcinogenic toxicity; Land use; Mineral resources: mineral resource scarcity; Fossil resources: fossil resource scarcity; Water consumption.

Considering the burden contributions from woven cotton textile, main contributions were dominant across ODP (56 – 62%), marine eutroph. (63%–83%), land use (61%–63%), and water consumption (32%–58%) across all the scenarios. N_2_O released from the cotton farming systems due to fertilization and irrigation was the main contributor to ODP with collateral damage observed across GWP due to an increase in electricity consumption attributed to intensive farming practices [41, 42]. Occupation and transformation of land for cotton cultivation as well as the use of nitrate fertilizers also produce land‐use and eutrophication hotspots [42]. 256 Gm^3^ of water is required yearly for cotton products worldwide [43], which contributes significantly to water consumption.

Contributions due to EMIM OAc utilization was dominant in freshwater eutrophication, ranging between 36% and 76%. EMIM OAc also shows high contributions to freshwater (11%–42%), terrestrial (15%–51%), marine (12%–45%) ecotoxicities, and human non‐carcino. (15%–50%). The high burdens brought by EMIM OAc across these impact categories were due to the use of 1‐ethylimidazole in the EMIM OAc production. The production of 1‐ethylimidazole results in high chemical oxygen demand (COD) due to emissions of phosphate and phosphorus, contributing to freshwater eutrophication. Metal emissions, especially zinc, copper, and arsenic, originated from the production of 1‐ethylimidazole due to the use of glyoxal and heat from steam, were the main hotspots in ecotoxicities in the biosphere and human health. Increasing the DMSO concentration saw an increase in burden contributions, particularly across PMF (16%), terrestrial acidification (15%) and human health (10%) mainly due to NO_x_, particulates and SO_2_ originating from the dimethyl carbonate production.

The recycling of EMIM OAc and DMSO from the wastewater was not considered in the present study because this was constrained by the coagulation and rinsing steps in the present ACC production process. The solvent became highly diluted in the wastewater (<0.4 wt.%). Recovering the solvent through evaporation or distillation would require an enormous amount of energy input to remove large quantity of water. The additional recovery required for the separation of EMIM OAc from DMSO in the solvent mixture would further increase the energy demand in the solvent recovery process. Water use and wastewater production in the ACC production (Figure 1) provided minimal contributions across all impact categories, with wastewater treatment used by the market providing environmental benefits within water consumption. However, there would be residual EMIM OAc and DMSO in the wastewater, which is a risk to the environment and human health. Toxicity and the biodegradability issues of imidazolium‐based ILs like EMIM OAc are recognizable [27, 44]. Imidazolium‐based ILs were reviewed to be barely biodegradable, suggesting that they can have a long residence time in the environment, and the aquatic lives would be directly exposed to the ILs in the open environment [27, 45]. Humans can be exposed to EMIM OAc through the drinking water route [27]. Although toxicity of EMIM OAc is acute with LD_50_ ∼ 2000 mg/kg [46], its long term effect to human health is unclear. Hormesis in both Gram‐positive and Gram‐negative bacteria can be induced by EMIM OAc, which poses threats to ecosystem balances [47]. DMSO is not inert and can induce changes in cellular processes and alterations in epigenetic landscape, posing a threat to human health [48]. Environmental burdens resulting from ACC production regarding the five solvents were also characterized using the ReCiPe 2016 endpoint Hierarchist perspective method. The details about the endpoint LCIA results regarding the five solvent scenarios are in Section S2 Tables S7–S11. The EMIM OAc endpoint LCIA result is in Section S2.1 Table S14. The characterized LCIA results of 1 kg EMIM OAc production across the endpoint impact categories are also presented in Section S2.1 Figure S1. Figure 6 shows the burdens across 3 endpoint impact categories and GWP from the midpoint impact analysis. The inventory contributions in the endpoint categories are similar to those observed in the midpoint categories. Electricity use was the main contributor, while the burden contributions from cellulose film and wastewater were negligible across human health, resource scarcity, and ecosystem quality. EMIM OAc also had a noticeable contribution to burdens in human health and resource scarcity due to the use of 1‐ethylimidazole in its production. The woven cotton textile had a significant role in ecosystem quality burdens.

**FIGURE 6:**
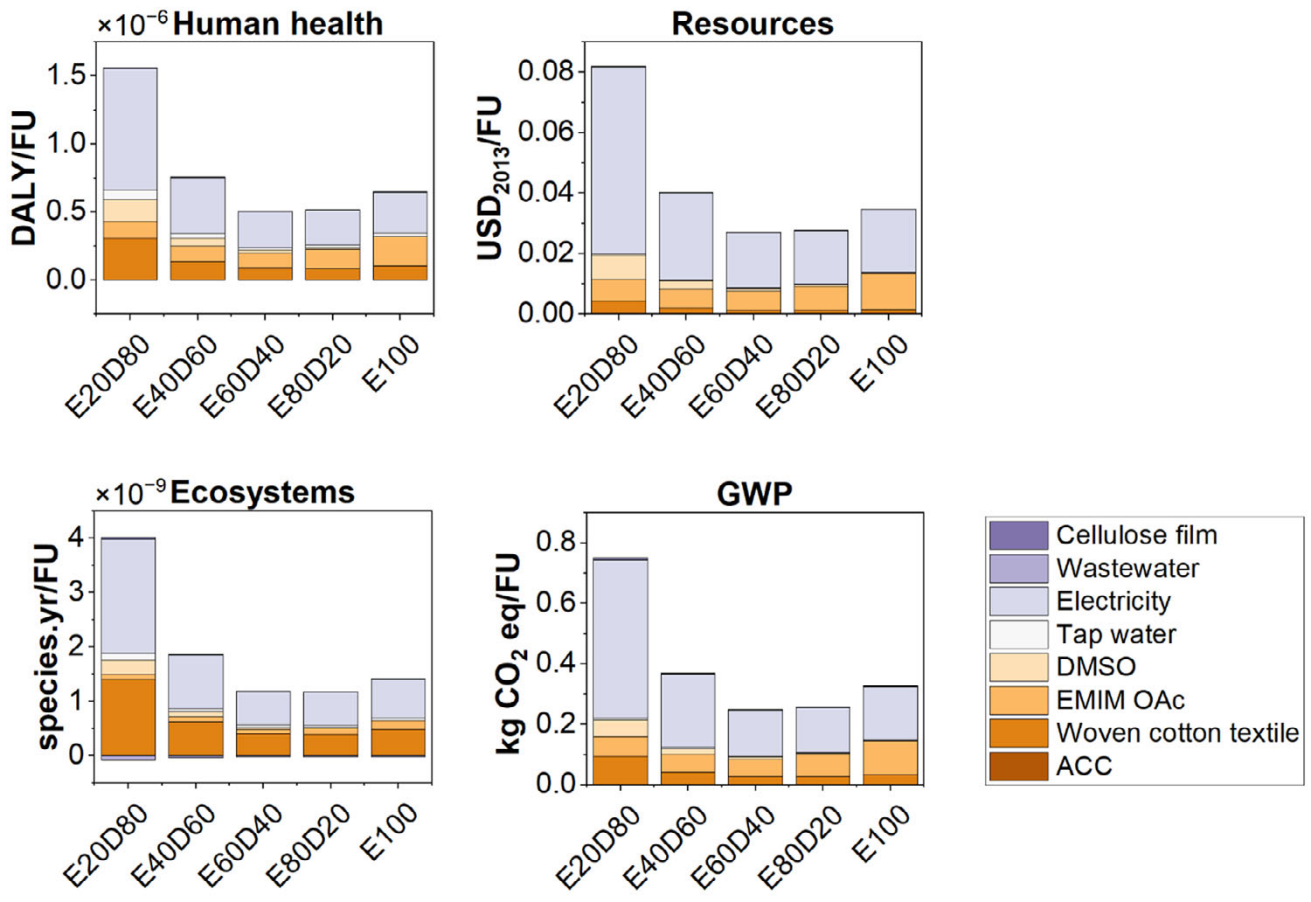
Characterized LCIA results for producing 1 functional unit of ACC produced in a lab scale in five solvents across endpoint impact categories using ReCiPe 2016 (H) method, and the midpoint impact category GWP (global warming potential). Resources: resource scarcity; Ecosystems: ecosystem quality.

The significant role of electricity usage in both midpoint and endpoint impact categories indicates that to reduce the environmental burdens, it would be more efficient to improve the ACC preparation method rather than changing the solvent composition. The amount of electricity consumption revealed in this study can be reduced by using a more energy efficient hot press or conducting cellulose dissolution and drying at a lower temperature.

#### 3.2.1 LCIA Comparison

There was no noticeable burden shifting between the midpoint and endpoint impact categories. The environmental burden from the BAU process (E100) was 6.49 × 10^−7^ DALY/FU, 1.39 × 10^−9^ species.yr/FU, 3.49 × 10^−2^ USD_2013_/FU, and 0.33 kg CO_2_ eq/FU in human health, ecosystems, resources, and GWP respectively. On examining all scenarios, the E60D40 system outperformed across human health, resources, and GWP, with respective burdens of 5.06 × 10^−7^ DALY/FU, 2.70 × 10^−2^ USD_2013_, and 0.25 kg CO_2_ eq/FU respectively. Its GWP was marginally lower than the E80D20 scenario by roughly 3%, while the E80D20 scenario produced 2% lower emissions across ecosystem quality (1.15 × 10^−9^ species.yr/FU). The E20D80 solvent mixture was the worst performing across all cases, producing environmental burdens in human health, ecosystems, resources, and GWP of 1.56 × 10^−6^ DALY/FU, 3.94 × 10^−9^ species.yr/FU, 8.20 × 10^−2^ USD_2013_/FU, and 0.75 kg CO_2_ eq/FU respectively. This was due to poorer mechanical properties than observed for the BAU scenario with an approximately 70% lower YM and thus requires more resources to maintain the same integrity as other standard ACC products.

Our results clearly show that reducing EMIM OAc usage lowers environmental burdens associated with human health, ecosystems, resources, and GWP, owing to the synergistic effects of DMSO addition. For instance, moving from E100 to E60D40 resulted in a notable 24% reduction in GWP. However, beyond the 40 wt.% DMSO benchmark, the LCA results deteriorate, indicating a possible DMSO constraint required for a more sustainable ACC production process.

### 3.3 Multiple Decision‐Making Criteria

To compare the environmental and mechanical performance of the solvent scenarios, a normalized spider plot (Figure 7) comprising multiple decision‐making criteria was constructed. The criteria considered were human health, ecosystem quality, resource scarcity, GWP, and YM, with each criterion assigned equal weighting. Each axis in the spider plot represented a distinct criterion. All criteria except YM represent environmental impacts and are intended to be minimized. YM was aimed to be maximized in this study. Therefore, it was constructed with a reverse score to be maximized. The ranking of solvent formulations was based on the sum of normalized scores, where the smallest aggregated normalized score indicates the best scenario, which has the lowest environmental impacts but a relatively high YM. In contrast, the highest aggregated normalized score indicates the worst‐case scenario, with the highest environmental impacts and a low YM. The ranking was interpreted within the maximum normalization method and equal weighting framework, because alternative normalization approaches or weighting schemes may influence the aggregated score outcomes and hence the ranking. The unnormalized and normalized scores of each criterion regarding each scenario are shown in Section S2.2 Tables S15 and S16, respectively.

**FIGURE 7:**
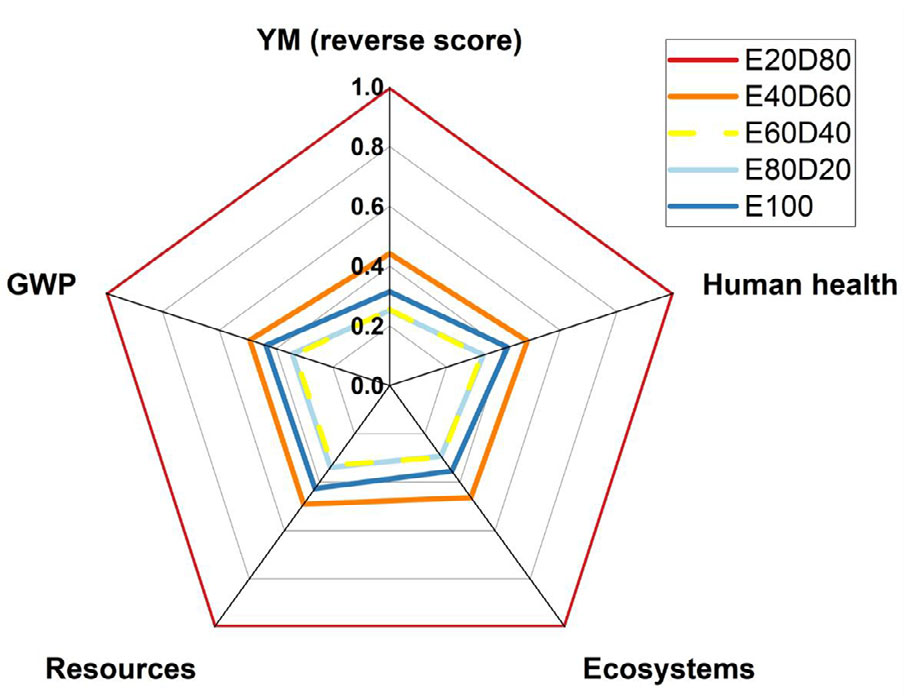
Sustainability analysis of the five investigated solvents used in the ACC lab production. The yellow dashed line indicates the best scenario that has the lowest environmental impact with a relatively high Young's modulus. YM: Young's modulus; GWP: global warming potential; Resources: resource scarcity; Ecosystems: ecosystem quality. Note that the ranking shown reflects the specific inventories and boundary conditions of this study and has not been validated through parameter‐sensitivity analysis.

The worst scenario was E20D80, which scored 1 in all criteria and thus performed the worst across all criteria. This directly aligns with the midpoint and endpoint impact analysis. Figure 7 also shows that the E60D40 scenario performed slightly better than the E80D20 scenario by about 1%. Under consideration of the cost of the solvents (EMIM OAc and DMSO), the benefit of using the E60D40 solvent in the ACC production is more significant than using the E80D20 solvent because the cost of EMIM OAc is much higher than the cost of DMSO. The cost of the DMSO in this study was £36/kg, while the cost of EMIM OAc was around £242/kg [49, 50]. Although the E60D40 scenario performed well in generating a relatively high YM with relatively low environmental impacts, it is possible for a global optimal solvent formulation to be found for better comparison and overall optimization of the ACC process. Thus, future work will focus on the design of experiment (DOE) to establish optimum ACC production conditions while also carrying out technoeconomic analysis (TEA) to understand economic sustainability in the ACC production. Regardless, our results illustrate the impact of appropriate solvent mixtures to provide synergistic interactions which promote both mechanical and environmental benefits.

## 4 Conclusions

This study evaluated the sustainability of ACCs produced in a laboratory scale using solvents that consist of EMIM OAc and DMSO in different concentrations (wt.%) with YM being the PI through both midpoint and endpoint impact categories. Under the ACC production conditions used in the present study without solvent recycling, the solvent composed of 60 wt.% EMIM OAc and 40 wt.% DMSO was the preferred solvent to use because it gave a relatively high stiffness with lower environmental impacts compared to the other four solvent compositions. Its resulting ACC had a GWP of 0.25 kg CO_2_ eq/FU and a YM of 5.3 ± 0.5 GPa.

The usage of high voltage electricity during the ACC production process was the main contributor to most of the environmental burdens. The high electricity was caused by the high temperature used for cellulose dissolution and drying in the ACC production. Therefore, it is important to improve the ACC preparation process either by using electricity from a cleaner source or using a process that does not require a high temperature or maximizing the production efficiency by scaling up the laboratory process. This study also revealed that the concentration of EMIM OAc in the solvent had a more significant role than the concentration of DMSO in environmental impacts. Hence, it is useful to find an ACC preparation method that requires less EMIM OAc as well. Ongoing research in the relationship between parameters controlled in the ACC production is required to discover the global optimal solvent mixture that consists of EMIM OAc and DMSO to be used in the ACC production to maximize YM. Therefore, we plan to do a DOE to establish optimum conditions for the ACC production process and to scale up the laboratory process. It will also be interesting to conduct a cradle‐to‐gate LCA of the ACC production based on other PIs, i.e., ultimate tensile strength, because YM alone does not represent the performance requirements for all structural applications of a material.

## Author Contributions

**Guanyi Chen**: conceptualization, investigation, methodology, validation, visualization, formal analysis, data curation, Writing – original draft. **Keeran Ward**: methodology, validation, formal analysis, data curation, supervision, writing – original draft, writing – review and editing, conceptualization. **Daniel L. Baker**: data curation, supervision, resources, writing – review and editing. **Diego Freire Ordóñez**: writing – review and editing, data curation, formal analysis, validation. **Michael E. Ries**: resources, project administration, writing – review and editing, writing – original draft, funding acquisition, conceptualization, supervision, methodology, validation, formal analysis.

## Funding

This work is supported by the EPSRC Centre for Doctoral Training in Soft Matter for Formulation and Industrial Innovation (SOFI2). Grant number: 2887677.

## Conflicts of Interest

The authors declare no conflicts of interest.

## Supporting information




**Supporting File 1**: gch270167‐sup‐0001‐SuppMat.docx.

**Supporting File 2**: gch270167‐sup‐0002‐SuppMat.xlsx.

## Data Availability

The data supporting this article have been included in the Supplementary Information. The Supplementary Information is openly available from the University of Leeds Data Repository: https://doi.org/10.5518/1883.
