# Supplementary material for: A Cradle‐to‐Gate Life Cycle Assessment of Sustainable All‐Cellulose Composites Using Imidazolium‐Based Ionic Liquid
Source: Glob Chall. 2026 Sep 30;10(10):e70167. doi: 10.1002/gch2.70167 (PMC13626338; doi:10.1002/gch2.70167)
Supplement: Supplementary file 1 [file GCH2-10-e70167-s002.docx]

Supporting Information

**A cradle-to-gate life cycle assessment of sustainable all-cellulose composites using imidazolium-based ionic liquid**

*Guanyi Chen^a,c^, Keeran Ward**^b^, Daniel L. Baker^a^, Diego Freire Ordóñez^d^, Michael E. Ries*^a^*

^a^School of Physics and Astronomy, University of Leeds, Leeds, LS2 9JT, UK

^b^School of Chemical and Process Engineering (SCAPE), University of Leeds, Leeds, LS2 9JT, UK

^c^School of Mathematics, University of Leeds, Leeds, LS2 9JT, UK

^d^Department of Chemical Engineering, Imperial College London, London, SW7 2AZ, UK.

*Corresponding author; Email: [M.E.Ries@leeds.ac.uk](mailto:M.E.Ries@leeds.ac.uk)

**Co-corresponding author; Email: [K.R.Ward@leeds.ac.uk](mailto:K.R.Ward@leeds.ac.uk)

# 1 Differential scanning calorimetry (DSC) of EMIM OAc

DSC experiments on 1-ethylimidazolium acetate (EMIM OAc) were performed to obtain its specific heat capacity at constant pressure. TZERO pans, TZERO Hermetic lids, and the Q2000 DSC machine were used. The DSC experiment had three cycles in total. The DSC experiment started with an equilibration of EMIM OAc at 5 ℃, followed by the heating cycle, and the cooling cycle interchangeably. During the heating cycle, EMIM OAc was heated from 5 ℃ to 195 ℃ at a rate of 10 ℃/min. During the cooling cycle, EMIM OAc was cooled from 195 ℃ to 5 ℃ at a rate of 10 ℃/min. The first heating and cooling cycles were used for equilibrating EMIM OAc. There was an isothermal stage of EMIM OAc at the end temperature of each cycle. The isothermal stage was 2 min. The data obtained from the second heating cycle was used for the estimation of the specific heat capacity equation of EMIM OAc. A second order polynomial was used to fit the data from 25 – 175 ℃. The specific heat capacity of EMIM OAc at 25 – 175 ℃ can be estimated by Equation S1, with *R^2^* (coefficient of determination) = 0.97.

$$C_{p\_EMIM OAc}(J/g\cdot℃)=1.66\times{10}^{-5}\mathcal{x}^{2}-5.29\times{10}^{-4}\mathcal{x}+1.71 (S1)$$

# 2 LCI and LCIA results overview

Table S1 shows the inventories of mass and energy balances for EMIM OAc, 1-ethylimidazole, and each solvent system used for the ACC production. The characterized impacts of ACCs produced in the five solvent systems calculated by ReCiPe midpoint and endpoint (H) 2016 are shown in Tables S2 – S11.

**Table S1.** LCI datasets obtained from the Ecoinvent v3.11 database.

| **Flow** | **System boundary** | **Ecoinvent entries** |
| --- | --- | --- |
| Glyoxal | 1-ethylimidazole | Glyoxal {RoW}\| market for glyoxal \| Cut-off, U |
| Formaldehyde | 1-ethylimidazole | Formaldehyde {RoW}\| market for formaldehyde \| Cut-off, U |
| Ammonia | 1-ethylimidazole | Ammonia, anhydrous, liquid {CN}\| market for ammonia, anhydrous, liquid \| Cut-off, U |
| Ethylamine | 1-ethylimidazole | Ethylamine {GLO}\| market for ethylamine \| Cut-off, U |
| Tap water | 1-ethylimidazole | Tap water {RoW}\| market for tap water \| Cut-off, U |
| Wastewater | 1-ethylimidazole | Wastewater, average {RoW}\| market for wastewater, average \| Cut-off, U |
| Heat from steam | 1-ethylimidazole | Heat, from steam, in chemical industry {RoW}\| market for heat, from steam, in chemical industry \| Cut-off, U |
| Heat from natural gas | 1-ethylimidazole | Heat, district or industrial, natural gas {RoW}\| market for heat, district or industrial, natural gas \| Cut-off, U |
| Electricity | 1-ethylimidazole | Electricity, high voltage {CN}\| market group for electricity, high voltage \| Cut-off, U |
| VOC | 1-ethylimidazole | VOC, volatile organic compounds as C |
| 1-ethylimidazole | EMIM OAc | Zhang et al.^[1]^ |
| Dimethyl carbonate | EMIM OAc | Dimethyl carbonate {GLO}\| market for dimethyl carbonate \| Cut-off, U |
| Acetic acid | EMIM OAc | Acetic acid {GLO}\| market for acetic acid \| Cut-off, U |
| Heat from natural gas | EMIM OAc | Heat, district or industrial, natural gas {RER}\| market group for heat, district or industrial, natural gas \| Cut-off, U |
| Electricity | EMIM OAc | Electricity, high voltage {AT}\| market for electricity, high voltage \| Cut-off, U |
| Woven cotton textile | E100, E80D20, E60D40, E40D60, E20D80 | Textile, woven cotton {GLO}\| market for textile, woven cotton \| Cut-off, U |
| EMIM OAc | E100, E80D20, E60D40, E40D60, E20D80 | Hand calculation and mass balances |
| Tap water | E100, E80D20, E60D40, E40D60, E20D80 | Tap water {GLO}\| market group for tap water \| Cut-off, U |
| Wastewater | E100, E80D20, E60D40, E40D60, E20D80 | Wastewater, average {Europe without Switzerland}\| market for wastewater, average \| Cut-off, U |
| Electricity | E100, E80D20, E60D40, E40D60, E20D80 | Electricity, high voltage {GB}\| market for electricity, high voltage \| Cut-off, U |
| DMSO | E80D20, E60D40, E40D60, E20D80 | Dimethyl sulfoxide {GLO}\| market for dimethyl sulfoxide \| Cut-off, U |

**Table S2.** E100 midpoint characterization (ReCiPe Midpoint H 2016). Functional unit (FU) = 2.8000 g ACC.

| Impact category | Unit | Total | ACC | Woven cotton textile | EMIM OAc | DMSO | Tap water | Electricity | Wastewater | Cellulose film |
| --- | --- | --- | --- | --- | --- | --- | --- | --- | --- | --- |
| Global warming | kg CO2 eq | 3.2738E-01 | 0.0000E+00 | 3.2508E-02 | 1.1225E-01 | 0.0000E+00 | 2.1253E-03 | 1.7730E-01 | 1.0025E-03 | 2.1921E-03 |
| Stratospheric ozone depletion | kg CFC11 eq | 3.3011E-07 | 0.0000E+00 | 1.8614E-07 | 4.0179E-08 | 0.0000E+00 | 1.2772E-09 | 9.7571E-08 | 4.0352E-09 | 9.0670E-10 |
| Ionizing radiation | kBq Co-60 eq | 1.1338E-01 | 0.0000E+00 | 1.6913E-03 | 2.8117E-03 | 0.0000E+00 | 2.2600E-04 | 1.0850E-01 | 1.5320E-04 | 0.0000E+00 |
| Ozone formation, Human health | kg NOx eq | 5.9630E-04 | 0.0000E+00 | 9.0217E-05 | 2.0116E-04 | 0.0000E+00 | 5.4224E-06 | 2.9366E-04 | 2.3444E-06 | 3.4996E-06 |
| Fine particulate matter formation | kg PM2.5 eq | 2.9888E-04 | 0.0000E+00 | 7.1578E-05 | 1.2434E-04 | 0.0000E+00 | 4.3360E-06 | 9.6095E-05 | 1.1633E-06 | 1.3711E-06 |
| Ozone formation, Terrestrial ecosystems | kg NOx eq | 6.2809E-04 | 0.0000E+00 | 9.2348E-05 | 2.1980E-04 | 0.0000E+00 | 5.5470E-06 | 3.0447E-04 | 2.4250E-06 | 3.5013E-06 |
| Terrestrial acidification | kg SO2 eq | 8.2267E-04 | 0.0000E+00 | 2.5784E-04 | 2.7769E-04 | 0.0000E+00 | 7.4661E-06 | 2.7204E-04 | 2.9653E-06 | 4.6604E-06 |
| Freshwater eutrophication | kg P eq | 2.4209E-04 | 0.0000E+00 | 2.8176E-05 | 1.8342E-04 | 0.0000E+00 | 1.2608E-06 | 2.3279E-05 | 5.9562E-06 | 9.2840E-11 |
| Marine eutrophication | kg N eq | 2.4610E-04 | 0.0000E+00 | 1.5603E-04 | 7.3300E-05 | 0.0000E+00 | 9.2949E-08 | 5.0917E-06 | 1.1582E-05 | 5.5380E-14 |
| Terrestrial ecotoxicity | kg 1,4-DCB | 4.1898E-01 | 0.0000E+00 | 5.5041E-02 | 2.1327E-01 | 0.0000E+00 | 4.1802E-03 | 1.3826E-01 | 3.8387E-03 | 4.3871E-03 |
| Freshwater ecotoxicity | kg 1,4-DCB | 9.9386E-03 | 0.0000E+00 | 2.4774E-03 | 4.1793E-03 | 0.0000E+00 | 1.3094E-04 | 3.0402E-03 | 1.1062E-04 | 1.9090E-07 |
| Marine ecotoxicity | kg 1,4-DCB | 1.2414E-02 | 0.0000E+00 | 2.3916E-03 | 5.5913E-03 | 0.0000E+00 | 1.8201E-04 | 4.0966E-03 | 1.4827E-04 | 3.7358E-06 |
| Human carcinogenic toxicity | kg 1,4-DCB | 4.3518E-02 | 0.0000E+00 | 3.9896E-03 | 1.7924E-02 | 0.0000E+00 | 3.8405E-03 | 1.7240E-02 | 5.2381E-04 | 7.3826E-09 |
| Human non-carcinogenic toxicity | kg 1,4-DCB | 2.9633E-01 | 0.0000E+00 | 5.0245E-02 | 1.5047E-01 | 0.0000E+00 | 2.8266E-03 | 8.3377E-02 | 9.1383E-03 | 2.6628E-04 |
| Land use | m2a crop eq | 3.5166E-02 | 0.0000E+00 | 2.1562E-02 | 1.7848E-03 | 0.0000E+00 | 4.9810E-05 | 1.1355E-02 | 2.8812E-05 | 3.8598E-04 |
| Mineral resource scarcity | kg Cu eq | 6.5130E-04 | 0.0000E+00 | 8.1291E-05 | 2.5564E-04 | 0.0000E+00 | 1.5967E-05 | 2.9060E-04 | 7.3883E-06 | 4.1946E-07 |
| Fossil resource scarcity | kg oil eq | 1.0808E-01 | 0.0000E+00 | 6.5683E-03 | 3.8707E-02 | 0.0000E+00 | 5.4666E-04 | 6.2062E-02 | 1.9372E-04 | 0.0000E+00 |
| Water consumption | m3 | -1.0665E-02 | 0.0000E+00 | 1.6118E-02 | -2.7705E-02 | 0.0000E+00 | 2.4981E-03 | 9.1778E-04 | -2.4934E-03 | 0.0000E+00 |

**Table S3.** E80D20 midpoint characterization (ReCiPe Midpoint H 2016). Functional unit (FU) = 2.2904 g ACC.

| Impact category | Unit | Total | ACC | Woven cotton textile | EMIM OAc | DMSO | Tap water | Electricity | Wastewater | Cellulose film |
| --- | --- | --- | --- | --- | --- | --- | --- | --- | --- | --- |
| Global warming | kg CO2 eq | 2.5835E-01 | 0.0000E+00 | 2.6719E-02 | 7.3970E-02 | 3.9367E-03 | 1.7896E-03 | 1.4930E-01 | 8.4414E-04 | 1.7931E-03 |
| Stratospheric ozone depletion | kg CFC11 eq | 2.6747E-07 | 0.0000E+00 | 1.5299E-07 | 2.6476E-08 | 6.2586E-10 | 1.0755E-09 | 8.2160E-08 | 3.3978E-09 | 7.4168E-10 |
| Ionizing radiation | kBq Co-60 eq | 9.5061E-02 | 0.0000E+00 | 1.3901E-03 | 1.8528E-03 | 1.3953E-04 | 1.9031E-04 | 9.1359E-02 | 1.2900E-04 | 0.0000E+00 |
| Ozone formation, Human health | kg NOx eq | 4.6977E-04 | 0.0000E+00 | 7.4150E-05 | 1.3256E-04 | 6.3792E-06 | 4.5660E-06 | 2.4728E-04 | 1.9741E-06 | 2.8627E-06 |
| Fine particulate matter formation | kg PM2.5 eq | 2.3514E-04 | 0.0000E+00 | 5.8830E-05 | 8.1935E-05 | 7.7081E-06 | 3.6512E-06 | 8.0917E-05 | 9.7955E-07 | 1.1216E-06 |
| Ozone formation, Terrestrial ecosystems | kg NOx eq | 4.9364E-04 | 0.0000E+00 | 7.5901E-05 | 1.4484E-04 | 6.9433E-06 | 4.6709E-06 | 2.5638E-04 | 2.0420E-06 | 2.8641E-06 |
| Terrestrial acidification | kg SO2 eq | 6.5804E-04 | 0.0000E+00 | 2.1192E-04 | 1.8299E-04 | 2.1455E-05 | 6.2869E-06 | 2.2908E-04 | 2.4970E-06 | 3.8122E-06 |
| Freshwater eutrophication | kg P eq | 1.7100E-04 | 0.0000E+00 | 2.3158E-05 | 1.2087E-04 | 1.2906E-06 | 1.0617E-06 | 1.9603E-05 | 5.0155E-06 | 7.5943E-11 |
| Marine eutrophication | kg N eq | 1.9074E-04 | 0.0000E+00 | 1.2825E-04 | 4.8302E-05 | 7.4036E-08 | 7.8269E-08 | 4.2875E-06 | 9.7525E-06 | 4.5301E-14 |
| Terrestrial ecotoxicity | kg 1,4-DCB | 3.1913E-01 | 0.0000E+00 | 4.5239E-02 | 1.4054E-01 | 6.5944E-03 | 3.5200E-03 | 1.1642E-01 | 3.2324E-03 | 3.5886E-03 |
| Freshwater ecotoxicity | kg 1,4-DCB | 7.7143E-03 | 0.0000E+00 | 2.0362E-03 | 2.7540E-03 | 1.6052E-04 | 1.1026E-04 | 2.5600E-03 | 9.3146E-05 | 1.5615E-07 |
| Marine ecotoxicity | kg 1,4-DCB | 9.5922E-03 | 0.0000E+00 | 1.9657E-03 | 3.6845E-03 | 2.1129E-04 | 1.5326E-04 | 3.4496E-03 | 1.2485E-04 | 3.0559E-06 |
| Human carcinogenic toxicity | kg 1,4-DCB | 3.3941E-02 | 0.0000E+00 | 3.2791E-03 | 1.1811E-02 | 6.5906E-04 | 3.2339E-03 | 1.4517E-02 | 4.4108E-04 | 6.0390E-09 |
| Human non-carcinogenic toxicity | kg 1,4-DCB | 2.2496E-01 | 0.0000E+00 | 4.1296E-02 | 9.9156E-02 | 4.0033E-03 | 2.3802E-03 | 7.0209E-02 | 7.6950E-03 | 2.1782E-04 |
| Land use | m2a crop eq | 2.8919E-02 | 0.0000E+00 | 1.7722E-02 | 1.1761E-03 | 7.7389E-05 | 4.1943E-05 | 9.5617E-03 | 2.4262E-05 | 3.1573E-04 |
| Mineral resource scarcity | kg Cu eq | 5.1131E-04 | 0.0000E+00 | 6.6814E-05 | 1.6846E-04 | 1.1330E-05 | 1.3445E-05 | 2.4470E-04 | 6.2214E-06 | 3.4312E-07 |
| Fossil resource scarcity | kg oil eq | 8.5665E-02 | 0.0000E+00 | 5.3985E-03 | 2.5506E-02 | 1.8764E-03 | 4.6032E-04 | 5.2260E-02 | 1.6313E-04 | 0.0000E+00 |
| Water consumption | m3 | -4.2050E-03 | 0.0000E+00 | 1.3247E-02 | -1.8257E-02 | 2.7508E-05 | 2.1036E-03 | 7.7282E-04 | -2.0996E-03 | 0.0000E+00 |

**Table S4.** E60D40 midpoint characterization (ReCiPe Midpoint H 2016). Functional unit (FU) = 2.3856 g ACC.

| Impact category | Unit | Total | ACC | Woven cotton textile | EMIM OAc | DMSO | Tap water | Electricity | Wastewater | Cellulose film |
| --- | --- | --- | --- | --- | --- | --- | --- | --- | --- | --- |
| Global warming | kg CO2 eq | 2.5028E-01 | 0.0000E+00 | 2.7236E-02 | 5.6629E-02 | 8.0262E-03 | 1.8437E-03 | 1.5381E-01 | 8.6963E-04 | 1.8676E-03 |
| Stratospheric ozone depletion | kg CFC11 eq | 2.6752E-07 | 0.0000E+00 | 1.5596E-07 | 2.0269E-08 | 1.2760E-09 | 1.1079E-09 | 8.4642E-08 | 3.5005E-09 | 7.7251E-10 |
| Ionizing radiation | kBq Co-60 eq | 9.7568E-02 | 0.0000E+00 | 1.4171E-03 | 1.4184E-03 | 2.8447E-04 | 1.9606E-04 | 9.4119E-02 | 1.3290E-04 | 0.0000E+00 |
| Ozone formation, Human health | kg NOx eq | 4.5454E-04 | 0.0000E+00 | 7.5587E-05 | 1.0148E-04 | 1.3006E-05 | 4.7039E-06 | 2.5475E-04 | 2.0338E-06 | 2.9817E-06 |
| Fine particulate matter formation | kg PM2.5 eq | 2.2771E-04 | 0.0000E+00 | 5.9970E-05 | 6.2726E-05 | 1.5716E-05 | 3.7615E-06 | 8.3361E-05 | 1.0091E-06 | 1.1682E-06 |
| Ozone formation, Terrestrial ecosystems | kg NOx eq | 4.7644E-04 | 0.0000E+00 | 7.7372E-05 | 1.1089E-04 | 1.4156E-05 | 4.8120E-06 | 2.6412E-04 | 2.1037E-06 | 2.9831E-06 |
| Terrestrial acidification | kg SO2 eq | 6.4888E-04 | 0.0000E+00 | 2.1603E-04 | 1.4009E-04 | 4.3743E-05 | 6.4768E-06 | 2.3600E-04 | 2.5724E-06 | 3.9706E-06 |
| Freshwater eutrophication | kg P eq | 1.4523E-04 | 0.0000E+00 | 2.3607E-05 | 9.2532E-05 | 2.6313E-06 | 1.0937E-06 | 2.0195E-05 | 5.1670E-06 | 7.9100E-11 |
| Marine eutrophication | kg N eq | 1.8240E-04 | 0.0000E+00 | 1.3073E-04 | 3.6978E-05 | 1.5095E-07 | 8.0633E-08 | 4.4170E-06 | 1.0047E-05 | 4.7184E-14 |
| Terrestrial ecotoxicity | kg 1,4-DCB | 2.9778E-01 | 0.0000E+00 | 4.6116E-02 | 1.0759E-01 | 1.3445E-02 | 3.6263E-03 | 1.1994E-01 | 3.3301E-03 | 3.7378E-03 |
| Freshwater ecotoxicity | kg 1,4-DCB | 7.3583E-03 | 0.0000E+00 | 2.0756E-03 | 2.1084E-03 | 3.2728E-04 | 1.1359E-04 | 2.6373E-03 | 9.5959E-05 | 1.6264E-07 |
| Marine ecotoxicity | kg 1,4-DCB | 9.0987E-03 | 0.0000E+00 | 2.0038E-03 | 2.8207E-03 | 4.3079E-04 | 1.5789E-04 | 3.5538E-03 | 1.2862E-04 | 3.1829E-06 |
| Human carcinogenic toxicity | kg 1,4-DCB | 3.2470E-02 | 0.0000E+00 | 3.3426E-03 | 9.0424E-03 | 1.3437E-03 | 3.3316E-03 | 1.4955E-02 | 4.5440E-04 | 6.2900E-09 |
| Human non-carcinogenic toxicity | kg 1,4-DCB | 2.0910E-01 | 0.0000E+00 | 4.2097E-02 | 7.5910E-02 | 8.1620E-03 | 2.4521E-03 | 7.2329E-02 | 7.9274E-03 | 2.2687E-04 |
| Land use | m2a crop eq | 2.9371E-02 | 0.0000E+00 | 1.8065E-02 | 9.0041E-04 | 1.5778E-04 | 4.3210E-05 | 9.8504E-03 | 2.4994E-05 | 3.2885E-04 |
| Mineral resource scarcity | kg Cu eq | 4.9288E-04 | 0.0000E+00 | 6.8108E-05 | 1.2896E-04 | 2.3099E-05 | 1.3851E-05 | 2.5209E-04 | 6.4093E-06 | 3.5738E-07 |
| Fossil resource scarcity | kg oil eq | 8.3336E-02 | 0.0000E+00 | 5.5031E-03 | 1.9527E-02 | 3.8257E-03 | 4.7423E-04 | 5.3838E-02 | 1.6805E-04 | 0.0000E+00 |
| Water consumption | m3 | 3.8374E-04 | 0.0000E+00 | 1.3504E-02 | -1.3977E-02 | 5.6084E-05 | 2.1671E-03 | 7.9616E-04 | -2.1630E-03 | 0.0000E+00 |

**Table S5.** E40D60 midpoint characterization (ReCiPe Midpoint H 2016). Functional unit (FU) = 3.4888 g ACC.

| Impact category | Unit | Total | ACC | Woven cotton textile | EMIM OAc | DMSO | Tap water | Electricity | Wastewater | Cellulose film |
| --- | --- | --- | --- | --- | --- | --- | --- | --- | --- | --- |
| Global warming | kg CO2 eq | 3.7032E-01 | 0.0000E+00 | 4.2651E-02 | 5.9226E-02 | 1.8854E-02 | 2.9078E-03 | 2.4258E-01 | 1.3715E-03 | 2.7313E-03 |
| Stratospheric ozone depletion | kg CFC11 eq | 4.1031E-07 | 0.0000E+00 | 2.4423E-07 | 2.1199E-08 | 2.9974E-09 | 1.7474E-09 | 1.3349E-07 | 5.5207E-09 | 1.1298E-09 |
| Ionizing radiation | kBq Co-60 eq | 1.5333E-01 | 0.0000E+00 | 2.2191E-03 | 1.4835E-03 | 6.6824E-04 | 3.0921E-04 | 1.4844E-01 | 2.0960E-04 | 0.0000E+00 |
| Ozone formation, Human health | kg NOx eq | 6.7181E-04 | 0.0000E+00 | 1.1837E-04 | 1.0614E-04 | 3.0552E-05 | 7.4187E-06 | 4.0177E-04 | 3.2075E-06 | 4.3605E-06 |
| Fine particulate matter formation | kg PM2.5 eq | 3.3714E-04 | 0.0000E+00 | 9.3912E-05 | 6.5603E-05 | 3.6916E-05 | 5.9324E-06 | 1.3147E-04 | 1.5916E-06 | 1.7084E-06 |
| Ozone formation, Terrestrial ecosystems | kg NOx eq | 7.0222E-04 | 0.0000E+00 | 1.2116E-04 | 1.1597E-04 | 3.3254E-05 | 7.5892E-06 | 4.1656E-04 | 3.3178E-06 | 4.3627E-06 |
| Terrestrial acidification | kg SO2 eq | 9.7984E-04 | 0.0000E+00 | 3.3829E-04 | 1.4651E-04 | 1.0275E-04 | 1.0215E-05 | 3.7220E-04 | 4.0570E-06 | 5.8068E-06 |
| Freshwater eutrophication | kg P eq | 1.8165E-04 | 0.0000E+00 | 3.6968E-05 | 9.6776E-05 | 6.1810E-06 | 1.7250E-06 | 3.1850E-05 | 8.1490E-06 | 1.1568E-10 |
| Marine eutrophication | kg N eq | 2.6669E-04 | 0.0000E+00 | 2.0472E-04 | 3.8674E-05 | 3.5458E-07 | 1.2717E-07 | 6.9662E-06 | 1.5846E-05 | 6.9003E-14 |
| Terrestrial ecotoxicity | kg 1,4-DCB | 4.2192E-01 | 0.0000E+00 | 7.2216E-02 | 1.1252E-01 | 3.1582E-02 | 5.7192E-03 | 1.8916E-01 | 5.2520E-03 | 5.4663E-03 |
| Freshwater ecotoxicity | kg 1,4-DCB | 1.0714E-02 | 0.0000E+00 | 3.2504E-03 | 2.2051E-03 | 7.6878E-04 | 1.7914E-04 | 4.1594E-03 | 1.5134E-04 | 2.3786E-07 |
| Marine ecotoxicity | kg 1,4-DCB | 1.3161E-02 | 0.0000E+00 | 3.1379E-03 | 2.9501E-03 | 1.0119E-03 | 2.4901E-04 | 5.6048E-03 | 2.0286E-04 | 4.6548E-06 |
| Human carcinogenic toxicity | kg 1,4-DCB | 4.7406E-02 | 0.0000E+00 | 5.2345E-03 | 9.4572E-03 | 3.1564E-03 | 5.2544E-03 | 2.3587E-02 | 7.1666E-04 | 9.1988E-09 |
| Human non-carcinogenic toxicity | kg 1,4-DCB | 2.9526E-01 | 0.0000E+00 | 6.5922E-02 | 7.9392E-02 | 1.9173E-02 | 3.8672E-03 | 1.1407E-01 | 1.2503E-02 | 3.3179E-04 |
| Land use | m2a crop eq | 4.5726E-02 | 0.0000E+00 | 2.8290E-02 | 9.4170E-04 | 3.7064E-04 | 6.8148E-05 | 1.5536E-02 | 3.9420E-05 | 4.8093E-04 |
| Mineral resource scarcity | kg Cu eq | 7.2585E-04 | 0.0000E+00 | 1.0666E-04 | 1.3488E-04 | 5.4260E-05 | 2.1845E-05 | 3.9758E-04 | 1.0108E-05 | 5.2265E-07 |
| Fossil resource scarcity | kg oil eq | 1.2395E-01 | 0.0000E+00 | 8.6178E-03 | 2.0422E-02 | 8.9867E-03 | 7.4792E-04 | 8.4911E-02 | 2.6504E-04 | 0.0000E+00 |
| Water consumption | m3 | 7.9232E-03 | 0.0000E+00 | 2.1147E-02 | -1.4618E-02 | 1.3174E-04 | 3.4178E-03 | 1.2557E-03 | -3.4114E-03 | 0.0000E+00 |

**Table S6.** E20D80 midpoint characterization (ReCiPe Midpoint H 2016). Functional unit (FU) = 8.1060 g ACC.

| Impact category | Unit | Total | ACC | Woven cotton textile | EMIM OAc | DMSO | Tap water | Electricity | Wastewater | Cellulose film |
| --- | --- | --- | --- | --- | --- | --- | --- | --- | --- | --- |
| Global warming | kg CO2 eq | 7.5159E-01 | 0.0000E+00 | 9.4496E-02 | 6.5260E-02 | 5.5569E-02 | 6.2420E-03 | 5.2073E-01 | 2.9442E-03 | 6.3460E-03 |
| Stratospheric ozone depletion | kg CFC11 eq | 8.7807E-07 | 0.0000E+00 | 5.4109E-07 | 2.3358E-08 | 8.8344E-09 | 3.7510E-09 | 2.8656E-07 | 1.1851E-08 | 2.6249E-09 |
| Ionizing radiation | kBq Co-60 eq | 3.2828E-01 | 0.0000E+00 | 4.9165E-03 | 1.6346E-03 | 1.9695E-03 | 6.6376E-04 | 3.1865E-01 | 4.4994E-04 | 0.0000E+00 |
| Ozone formation, Human health | kg NOx eq | 1.3646E-03 | 0.0000E+00 | 2.6225E-04 | 1.1695E-04 | 9.0047E-05 | 1.5925E-05 | 8.6246E-04 | 6.8855E-06 | 1.0131E-05 |
| Fine particulate matter formation | kg PM2.5 eq | 6.9150E-04 | 0.0000E+00 | 2.0806E-04 | 7.2286E-05 | 1.0881E-04 | 1.2735E-05 | 2.8223E-04 | 3.4165E-06 | 3.9693E-06 |
| Ozone formation, Terrestrial ecosystems | kg NOx eq | 1.4220E-03 | 0.0000E+00 | 2.6844E-04 | 1.2779E-04 | 9.8010E-05 | 1.6291E-05 | 8.9421E-04 | 7.1222E-06 | 1.0136E-05 |
| Terrestrial acidification | kg SO2 eq | 2.0569E-03 | 0.0000E+00 | 7.4950E-04 | 1.6144E-04 | 3.0285E-04 | 2.1928E-05 | 7.9898E-04 | 8.7090E-06 | 1.3492E-05 |
| Freshwater eutrophication | kg P eq | 2.9632E-04 | 0.0000E+00 | 8.1903E-05 | 1.0663E-04 | 1.8218E-05 | 3.7029E-06 | 6.8370E-05 | 1.7493E-05 | 2.6877E-10 |
| Marine eutrophication | kg N eq | 5.4647E-04 | 0.0000E+00 | 4.5357E-04 | 4.2614E-05 | 1.0451E-06 | 2.7299E-07 | 1.4954E-05 | 3.4015E-05 | 1.6032E-13 |
| Terrestrial ecotoxicity | kg 1,4-DCB | 8.1938E-01 | 0.0000E+00 | 1.6000E-01 | 1.2399E-01 | 9.3084E-02 | 1.2277E-02 | 4.0606E-01 | 1.1274E-02 | 1.2701E-02 |
| Freshwater ecotoxicity | kg 1,4-DCB | 2.1536E-02 | 0.0000E+00 | 7.2014E-03 | 2.4297E-03 | 2.2659E-03 | 3.8456E-04 | 8.9288E-03 | 3.2488E-04 | 5.5265E-07 |
| Marine ecotoxicity | kg 1,4-DCB | 2.6198E-02 | 0.0000E+00 | 6.9521E-03 | 3.2506E-03 | 2.9826E-03 | 5.3455E-04 | 1.2032E-02 | 4.3546E-04 | 1.0815E-05 |
| Human carcinogenic toxicity | kg 1,4-DCB | 9.4771E-02 | 0.0000E+00 | 1.1597E-02 | 1.0421E-02 | 9.3030E-03 | 1.1279E-02 | 5.0632E-02 | 1.5384E-03 | 2.1373E-08 |
| Human non-carcinogenic toxicity | kg 1,4-DCB | 5.7083E-01 | 0.0000E+00 | 1.4605E-01 | 8.7480E-02 | 5.6509E-02 | 8.3016E-03 | 2.4488E-01 | 2.6839E-02 | 7.7088E-04 |
| Land use | m2a crop eq | 9.9504E-02 | 0.0000E+00 | 6.2677E-02 | 1.0376E-03 | 1.0924E-03 | 1.4629E-04 | 3.3349E-02 | 8.4620E-05 | 1.1174E-03 |
| Mineral resource scarcity | kg Cu eq | 1.4681E-03 | 0.0000E+00 | 2.3630E-04 | 1.4862E-04 | 1.5992E-04 | 4.6894E-05 | 8.5347E-04 | 2.1699E-05 | 1.2143E-06 |
| Fossil resource scarcity | kg oil eq | 2.5253E-01 | 0.0000E+00 | 1.9093E-02 | 2.2503E-02 | 2.6487E-02 | 1.6055E-03 | 1.8227E-01 | 5.6896E-04 | 0.0000E+00 |
| Water consumption | m3 | 3.3843E-02 | 0.0000E+00 | 4.6852E-02 | -1.6107E-02 | 3.8829E-04 | 7.3369E-03 | 2.6955E-03 | -7.3230E-03 | 0.0000E+00 |

**Table S7.** E100 endpoint characterization (ReCiPe Endpoint H 2016). Functional unit (FU) = 2.8000 g ACC.

| Damage category | Unit | Total | ACC | Woven cotton textile | EMIM OAc | DMSO | Tap water | Electricity | Wastewater | Cellulose film |
| --- | --- | --- | --- | --- | --- | --- | --- | --- | --- | --- |
| Human health | DALY | 6.4934E-07 | 0.0000E+00 | 1.0543E-07 | 2.1403E-07 | 0.0000E+00 | 2.3260E-08 | 3.0371E-07 | -5.1221E-11 | 2.9596E-09 |
| Ecosystems | species.yr | 1.3945E-09 | 0.0000E+00 | 4.8065E-10 | 1.6987E-10 | 0.0000E+00 | 4.1985E-11 | 7.1654E-10 | -2.5552E-11 | 1.1051E-11 |
| Resources | USD2013 | 3.4888E-02 | 0.0000E+00 | 1.5060E-03 | 1.2146E-02 | 0.0000E+00 | 1.1501E-04 | 2.1062E-02 | 5.9332E-05 | 9.6784E-08 |

**Table S8.** E80D20 endpoint characterization (ReCiPe Endpoint H 2016). Functional unit (FU) = 2.2904 g ACC.

| Damage category | Unit | Total | ACC | Woven cotton textile | EMIM OAc | DMSO | Tap water | Electricity | Wastewater | Cellulose film |
| --- | --- | --- | --- | --- | --- | --- | --- | --- | --- | --- |
| Human health | DALY | 5.1704E-07 | 0.0000E+00 | 8.6654E-08 | 1.4104E-07 | 1.1648E-08 | 1.9586E-08 | 2.5574E-07 | -4.3131E-11 | 2.4210E-09 |
| Ecosystems | species.yr | 1.1517E-09 | 0.0000E+00 | 3.9505E-10 | 1.1194E-10 | 1.8520E-11 | 3.5353E-11 | 6.0336E-10 | -2.1517E-11 | 9.0395E-12 |
| Resources | USD2013 | 2.7701E-02 | 0.0000E+00 | 1.2378E-03 | 8.0035E-03 | 5.7741E-04 | 9.6847E-05 | 1.7736E-02 | 4.9961E-05 | 7.9169E-08 |

**Table S9.** E60D40 endpoint characterization (ReCiPe Endpoint H 2016). Functional unit (FU) = 2.3856 g ACC.

| Damage category | Unit | Total | ACC | Woven cotton textile | EMIM OAc | DMSO | Tap water | Electricity | Wastewater | Cellulose film |
| --- | --- | --- | --- | --- | --- | --- | --- | --- | --- | --- |
| Human health | DALY | 5.0618E-07 | 0.0000E+00 | 8.8333E-08 | 1.0797E-07 | 2.3749E-08 | 2.0178E-08 | 2.6347E-07 | -4.4433E-11 | 2.5216E-09 |
| Ecosystems | species.yr | 1.1714E-09 | 0.0000E+00 | 4.0270E-10 | 8.5697E-11 | 3.7759E-11 | 3.6421E-11 | 6.2158E-10 | -2.2166E-11 | 9.4153E-12 |
| Resources | USD2013 | 2.6989E-02 | 0.0000E+00 | 1.2618E-03 | 6.1272E-03 | 1.1772E-03 | 9.9772E-05 | 1.8271E-02 | 5.1470E-05 | 8.2460E-08 |

**Table S10.** E40D60 endpoint characterization (ReCiPe Endpoint H 2016). Functional unit (FU) = 3.4888 g ACC.

| Damage category | Unit | Total | ACC | Woven cotton textile | EMIM OAc | DMSO | Tap water | Electricity | Wastewater | Cellulose film |
| --- | --- | --- | --- | --- | --- | --- | --- | --- | --- | --- |
| Human health | DALY | 7.5800E-07 | 0.0000E+00 | 1.3833E-07 | 1.1293E-07 | 5.5786E-08 | 3.1823E-08 | 4.1552E-07 | -7.0078E-11 | 3.6877E-09 |
| Ecosystems | species.yr | 1.8255E-09 | 0.0000E+00 | 6.3062E-10 | 8.9627E-11 | 8.8696E-11 | 5.7441E-11 | 9.8031E-10 | -3.4960E-11 | 1.3769E-11 |
| Resources | USD2013 | 4.0204E-02 | 0.0000E+00 | 1.9759E-03 | 6.4082E-03 | 2.7653E-03 | 1.5735E-04 | 2.8816E-02 | 8.1176E-05 | 1.2059E-07 |

**Table S11.** E20D80 endpoint characterization (ReCiPe Endpoint H 2016). Functional unit (FU) = 8.1060 g ACC.

| Damage category | Unit | Total | ACC | Woven cotton textile | EMIM OAc | DMSO | Tap water | Electricity | Wastewater | Cellulose film |
| --- | --- | --- | --- | --- | --- | --- | --- | --- | --- | --- |
| Human health | DALY | 1.5640E-06 | 0.0000E+00 | 3.0647E-07 | 1.2443E-07 | 1.6442E-07 | 6.8313E-08 | 8.9198E-07 | -1.5043E-10 | 8.5681E-09 |
| Ecosystems | species.yr | 3.9420E-09 | 0.0000E+00 | 1.3972E-09 | 9.8758E-11 | 2.6142E-10 | 1.2331E-10 | 2.1044E-09 | -7.5046E-11 | 3.1992E-11 |
| Resources | USD2013 | 8.1960E-02 | 0.0000E+00 | 4.3776E-03 | 7.0610E-03 | 8.1505E-03 | 3.3779E-04 | 6.1859E-02 | 1.7426E-04 | 2.8019E-07 |

## 2.1 LCI and LCIA of EMIM OAc

Table S12 presents the inventories of producing 1 kg EMIM OAc followed the CBILS process. Table S12 – S14 show the characterized impacts of EMIM OAc using ReCiPe midpoint and endpoint H 2016.

**Table S12.** LCI of EMIM OAc. Functional unit (FU) = 1kg EMIM OAc.

| **Inputs-materials** | |
| --- | --- |
| 1-ethylimidazole [g] | 564.7700 |
| Dimethyl carbonate [g] | 529.2300 |
| Acetic acid [g] | 352.8000 |
| **Inputs-energy** | |
| Heat [kJ] | 6747.3600 |
| Electricity [kWh] | 0.1190 |
| **Outputs-materials** | |
| EMIM OAc [kg] | 1.0000 |
| **Outputs-emissions** | |
| Carbon dioxide [g] | 258.5600 |

**Table S13.** EMIM OAc midpoint characterization (ReCiPe Midpoint H 2016). Functional unit (FU) = 1kg EMIM OAc.

| Impact category | Unit | Total | EMIM OAc | 1-ethylimidazole | Dimethyl carbonate | Acetic acid | Heat from natural gas | Electricity |
| --- | --- | --- | --- | --- | --- | --- | --- | --- |
| Global warming | kg CO2 eq | 1.1513E+01 | 2.5856E-01 | 8.2511E+00 | 1.6569E+00 | 9.3936E-01 | 3.7798E-01 | 2.9164E-02 |
| Stratospheric ozone depletion | kg CFC11 eq | 4.1209E-06 | 0.0000E+00 | 3.6115E-06 | 3.0852E-07 | 1.3158E-07 | 5.1865E-08 | 1.7387E-08 |
| Ionizing radiation | kBq Co-60 eq | 2.8838E-01 | 0.0000E+00 | 1.8775E-01 | 7.1759E-02 | 1.9520E-02 | 2.2885E-03 | 7.0547E-03 |
| Ozone formation, Human health | kg NOx eq | 2.0632E-02 | 0.0000E+00 | 1.5276E-02 | 3.3795E-03 | 1.5695E-03 | 3.6563E-04 | 4.1033E-05 |
| Fine particulate matter formation | kg PM2.5 eq | 1.2753E-02 | 0.0000E+00 | 9.8573E-03 | 1.9417E-03 | 8.5471E-04 | 7.8363E-05 | 2.0627E-05 |
| Ozone formation, Terrestrial ecosystems | kg NOx eq | 2.2544E-02 | 0.0000E+00 | 1.6562E-02 | 3.7855E-03 | 1.7499E-03 | 4.0406E-04 | 4.2500E-05 |
| Terrestrial acidification | kg SO2 eq | 2.8481E-02 | 0.0000E+00 | 2.2644E-02 | 3.7143E-03 | 1.8619E-03 | 2.0310E-04 | 5.7974E-05 |
| Freshwater eutrophication | kg P eq | 1.8812E-02 | 0.0000E+00 | 1.7939E-02 | 5.8128E-04 | 2.5166E-04 | 9.6816E-06 | 3.0605E-05 |
| Marine eutrophication | kg N eq | 7.5179E-03 | 0.0000E+00 | 7.4588E-03 | 4.0128E-05 | 1.3284E-05 | 3.6491E-06 | 2.0546E-06 |
| Terrestrial ecotoxicity | kg 1,4-DCB | 2.1874E+01 | 0.0000E+00 | 1.5760E+01 | 4.0422E+00 | 1.9977E+00 | 5.8046E-02 | 1.6245E-02 |
| Freshwater ecotoxicity | kg 1,4-DCB | 4.2865E-01 | 0.0000E+00 | 3.1324E-01 | 8.3995E-02 | 2.8548E-02 | 1.7607E-03 | 1.1101E-03 |
| Marine ecotoxicity | kg 1,4-DCB | 5.7346E-01 | 0.0000E+00 | 4.2210E-01 | 1.0941E-01 | 3.7943E-02 | 2.5108E-03 | 1.4980E-03 |
| Human carcinogenic toxicity | kg 1,4-DCB | 1.8384E+00 | 0.0000E+00 | 1.3859E+00 | 3.0226E-01 | 1.2682E-01 | 1.9419E-02 | 4.0029E-03 |
| Human non-carcinogenic toxicity | kg 1,4-DCB | 1.5433E+01 | 0.0000E+00 | 1.2564E+01 | 1.9895E+00 | 8.0688E-01 | 3.6465E-02 | 3.6587E-02 |
| Land use | m2a crop eq | 1.8306E-01 | 0.0000E+00 | 1.2903E-01 | 3.6470E-02 | 1.5054E-02 | 1.4486E-03 | 1.0592E-03 |
| Mineral resource scarcity | kg Cu eq | 2.6219E-02 | 0.0000E+00 | 1.8281E-02 | 5.6813E-03 | 1.9685E-03 | 2.4929E-04 | 3.9456E-05 |
| Fossil resource scarcity | kg oil eq | 3.9699E+00 | 0.0000E+00 | 2.7491E+00 | 6.7406E-01 | 3.9270E-01 | 1.4628E-01 | 7.7391E-03 |
| Water consumption | m3 | -2.8415E+00 | 0.0000E+00 | -2.8618E+00 | 1.1729E-02 | 7.5706E-03 | 3.4011E-04 | 6.0596E-04 |

**Table S14.** EMIM OAc endpoint characterization (ReCiPe Endpoint H 2016). Functional unit (FU) = 1kg EMIM OAc.

| Damage category | Unit | Total | EMIM OAc | 1-ethylimidazole | Dimethyl carbonate | Acetic acid | Heat from natural gas | Electricity |
| --- | --- | --- | --- | --- | --- | --- | --- | --- |
| Human health | DALY | 2.1952E-05 | 2.3994E-07 | 1.4910E-05 | 4.2375E-06 | 2.0287E-06 | 4.7388E-07 | 6.1933E-08 |
| Ecosystems | species.yr | 1.7423E-08 | 7.2399E-10 | 4.8397E-09 | 6.8659E-09 | 3.6821E-09 | 1.1792E-09 | 1.3186E-10 |
| Resources | USD2013 | 1.2457E+00 | 0.0000E+00 | 8.4799E-01 | 2.2067E-01 | 1.2310E-01 | 5.2350E-02 | 1.5968E-03 |

Figure S1 presents the LCIA results of the synthesis of 1 kg EMIM OAc in the simulated CBILS process. ^[4]^ Burdens associated with electricity were negligible, which is consistent with the literature, as the CBILS process is efficient, with a conversion rate > 99%. ^[4]^ The environmental burdens from the EMIM OAc production were 11.51 kg CO_2_ eq/kg EMIM OAc, 2.2 x 10^-5^ DALY/kg EMIM OAc, 1.25 USD_2013_/kg EMIM OAc, and 1.74 x 10^-8^ species.yr/kg EMIM OAc in GWP, human health, resources and ecosystems respectively. 1-ethylimidazole was the main contributor in GWP, human health and resources, while dimethyl carbonate (DMC) was the major contributor in ecosystems.

The contribution from each inventory was similar to the results reviewed by Ordóñez et al. ^[3]^ However, our GWP of the EMIM OAc production was much higher, and the main contributor in ecosystems was DMC instead of 1-ethylimidazole in our study. The total GWP of the EMIM OAc synthesis reviewed in Ordóñez et al. ^[3]^ was 4.6 kg CO_2_ eq/kg EMIM OAc, which was about 2.5 times lower than our result. 1-ethylimidazole contributed about 8.29 kg CO_2_ eq/kg EMIM OAc in our study, while its contribution was about 2.76 kg CO_2_ eq/kg EMIM OAc in Ordóñez et al.. ^[3]^

**Figure S1.** Characterized LCIA results of 1kg EMIM OAc production across endpoint impact categories (human health, resource scarcity, ecosystem quality), and global warming potential (GWP). Resources: resource scarcity; Ecosystems: ecosystem quality.

The high contribution of 1-ethylimidazole to GWP, human health and resources was driven by its production, which relied on heat and electricity generated mainly from fossil fuel combustion in Mainland China- mainly attributed to coal. Burdens linked to CO_2_ N_2_O and CH_4_ fugitive emissions, particulates and SO_2_ during the combustion of fossil fuels and the production of glyoxal were noticeable hotspots across GWP and human health. The use of fossil fuels in energy production places burdens on resources as well since it is non-renewable. The high contribution of DMC to ecosystems was due to its production using hydropower and fossil fuels, which had a high impact on ecosystems. Therefore, to reduce such impacts, cleaner supply chains utilizing renewable/low carbon energy sources and feedstocks are needed.

## 2.2 Multiple decision-making criteria

Tables S15-S16 give unnormalized and normalized scores (in 2 decimal places) of decision-making criteria used in section 4.3. The decision-making criteria are human health, ecosystem quality, resource scarcity, GWP, and YM (reverse score). For all the criteria, the scores were normalized by the highest unnormalized score across the scenarios, using the maximum normalization method ^[5]^ explained in Section 2.5.4 in the main text. In the present study, the highest unnormalized score across the scenarios for each criterion was E20D80.

**Table S15.** Unnormalized scores of decision-making criteria regarding the five solvents to 2 decimal places.

| Criteria | E20D80 | E40D60 | E60D40 | E80D20 | E100 |
| --- | --- | --- | --- | --- | --- |
| Human health | 1.56E-06 | 7.58E-07 | 5.06E-07 | 5.17E-07 | 6.49E-07 |
| Ecosystem quality | 3.94E-09 | 1.83E-09 | 1.17E-09 | 1.15E-09 | 1.39E-09 |
| Resource scarcity | 8.20E-02 | 4.02E-02 | 2.70E-02 | 2.77E-02 | 3.49E-02 |
| GWP | 7.52E-01 | 3.70E-01 | 2.50E-01 | 2.58E-01 | 3.27E-01 |
| YM (reverse score) | 7.45E-01 | 3.29E-01 | 1.89E-01 | 1.87E-01 | 2.34E-01 |

**Table S16.** Normalized scores of decision-making criteria regarding the five solvents to 2 decimal places.

| Criteria | E20D80 | E40D60 | E60D40 | E80D20 | E100 |
| --- | --- | --- | --- | --- | --- |
| Human health | 1.00 | 0.48 | 0.32 | 0.33 | 0.42 |
| Ecosystem quality | 1.00 | 0.46 | 0.30 | 0.29 | 0.35 |
| Resource scarcity | 1.00 | 0.49 | 0.33 | 0.34 | 0.43 |
| GWP | 1.00 | 0.49 | 0.33 | 0.34 | 0.44 |
| YM (reverse score) | 1.00 | 0.44 | 0.25 | 0.25 | 0.31 |

# References

1.	Zhang, Y., Liu, F., Zhang, J., Gao, W., Yu, J., & Wang, L. (2024). Life Cycle Thinking-Based GREENNESS Framework for Advancing Green Chemistry: Case Study with Typical Ionic Liquids for Cellulose Dissolution and Regeneration. *ACS Sustainable Chemistry and Engineering*, *12*(16), 6400–6411. https://doi.org/10.1021/acssuschemeng.4c00610

2.	*cellophane | GLAD*. (n.d.). Retrieved 20 June 2026, from https://www.globallcadataaccess.org/cellophane

3.	Ordóñez, D. F., Pontillo, A. R. N., Mac Dowell, N., Welton, T., & Lee, K. Y. (2025). Unveiling the environmental costs of lignocellulosic film production with ionic liquids: the case of 1-ethyl-3-methylimidazolium acetate. *RSC Sustainability*, *3*(7), 3002–3008. <https://doi.org/10.1039/d5su00212e>

4.	Kalb, R. S., Stepurko, E. N., Emel’yanenko, V. N., & Verevkin, S. P. (2016). Carbonate based ionic liquid synthesis (CBILS®): thermodynamic analysis. *Physical Chemistry Chemical Physics*, *18*(46), 31904–31913. <https://doi.org/10.1039/c6cp06594e>

5.	Sałabun, W., Watróbski, J., & Shekhovtsov, A. (2020). Are MCDA Methods Benchmarkable? A Comparative Study of TOPSIS, VIKOR, COPRAS, and PROMETHEE II Methods. *Symmetry 2020, Vol. 12, Page 1549*, *12*(9), 1549. https://doi.org/10.3390/SYM12091549
